# First occurrence of a herrerasaurid dinosaur in a traversodontid cynodont-dominated Late Triassic fauna of southern Brazil

**DOI:** 10.1007/s00114-026-02185-1

**Published:** 2026-10-19

**Authors:** Rodrigo Temp Müller

**Affiliations:** Centro de Apoio à Pesquisa Paleontológica, Universidade Federal de Santa Maria, Rua Maximiliano Vizzotto, 598, São João do Polêsine, 97230-000 RS Brazil

**Keywords:** Carnian, Dinosauria, Herrerasauria, Herrerasauridae, Saurischia

## Abstract

Herrerasauridae comprises the oldest large-bodied predatory dinosaurs and is currently known exclusively from the Carnian (Late Triassic) of South America. In both Argentina and Brazil, herrerasaurid occurrences have previously been restricted to rhynchosaur-dominated assemblages, with no records from younger strata characterized by the abundance of the traversodontid cynodont *Exaeretodon*. Here, I describe CAPPA/UFSM 0539, a partial left pubis recovered from the *Exaeretodon*-dominated strata of the *Hyperodapedon* Assemblage Zone (AZ) of southern Brazil. The specimen preserves a combination of pubic features that is currently unique to herrerasaurids, including a well-developed pubic boot with anterior and posterior distal expansions. A phylogenetic analysis further supports its referral to an indeterminate herrerasaurid. This specimen represents the first record of Herrerasauridae from the *Exaeretodon*-dominated strata, demonstrating that members of the clade persisted despite the faunal turnover associated with the decline of rhynchosaurs and the increasing abundance of *Exaeretodon*. The occurrence expands the known diversity of large predators in these ecosystems and suggests that herrerasaurids retained the ecological plasticity to occupy apex predatory roles under changing community compositions. If the Brazilian *Exaeretodon*-dominated strata are younger than the *Hyperodapedon* Acme Zone, CAPPA/UFSM 0539 may also represent the youngest known record of Herrerasauridae worldwide.

**Supplementary Information:**

The online version contains supplementary material available at https://doi.org/10.1007/s00114-026-02185-1.

## Introduction

Herrerasaurid dinosaurs were the oldest large predatory dinosaurs, reaching body lengths of over 5 m (Novas 1986; Langer et al. 2010; Garcia et al. 2021). All known herrerasaurids are characterized by a relatively robust skull bearing ziphodont teeth, forelimbs equipped with large trenchant manual unguals, a perforate acetabulum, and an obligate bipedal posture (Sereno 1994; Sereno and Novas 1994; Langer et al. 2010; Novas et al. 2021). The phylogenetic history of the clade has been marked by a series of competing hypotheses. Over the years, herrerasaurids have been interpreted as lying outside Dinosauria (Gauthier and Padian 1985; Novas 1992; Fraser et al. 2002; Baron and Williams 2018), as theropod dinosaurs (Sereno and Novas 1992; Novas 1996; Rauhut 2003; Nesbitt et al. 2009; Martínez et al. 2011; Sues et al. 2011), as the sister group to Sauropodomorpha (Baron et al. 2017), and as early-diverging saurischians outside Eusaurischia (Langer and Benton 2006; Irmis et al. 2007; Ezcurra 2010; Müller and Garcia 2020a; Novas et al. 2021; Garcia et al. 2024; Ezcurra et al. 2025). The most comprehensive and recent phylogenetic analyses have generally recovered the clade within Saurischia as an early-diverging lineage, basal to the Theropoda-Sauropodomorpha split and belonging to the more inclusive clade Herrerasauria (Novas et al. 2021; Garcia et al. 2024; Ezcurra et al. 2025; Srivastava and Nesbitt 2026).

The clade Herrerasauridae is currently represented by four valid species from the Carnian (Late Triassic) of South America: *Herrerasaurus ischigualastensis* Reing 1963 and *Sanjuansaurus gordilloi* Martínez & Alcober 2010; from the Ischigualasto Formation of Argentina (Reig 1963; Alcober and Martínez 2010), and *Staurikosaurus pricei* Colbert 1970 and *Gnathovorax cabrairai* Pacheco et al. 2019; from the Candelária Sequence of southern Brazil (Colbert 1970; Pacheco et al. 2019). In addition, closely related members of the more inclusive clade Herrerasauria have been reported from younger Upper Triassic deposits (Nesbitt et al. 2009; Sues et al. 2011; Ezcurra et al. 2025; Srivastava and Nesbitt 2026). According to the fossil record, neither herrerasaurids nor herrerasaurians survived into the Jurassic, when they were likely replaced by theropod dinosaurs as the dominant large terrestrial predators (Pacheco et al. 2019).

Although the fossil record of Herrerasauridae is relatively abundant in Argentina (Reig 1963; Novas 1986, 1994; Sereno 1994; Sereno and Novas 1994; Alcober and Martínez 2010; Martínez et al. 2013; Novas et al. 2021), the group remains considerably less common in Brazil (Müller In press). Nevertheless, the Brazilian record includes exceptionally well-preserved specimens, including complete skeletons (Pacheco et al. 2019). In both Argentina and Brazil, the fossil record of herrerasaurids is restricted to strata characterized by the abundance of rhynchosaurs (Martínez et al. 2013; Schultz et al. 2020). In the Ischigualasto Formation of Argentina, herrerasaurid occurrences are confined to the *Scaphonyx*-*Exaeretodon*-*Herrerasaurus* Biozone (Martínez et al. 2013), whereas in the Candelária Sequence of southern Brazil they are restricted to the *Hyperodapedon* Acme Zone of the *Hyperodapedon* Assemblage Zone (AZ; Colbert 1970; Pacheco et al. 2019; Schultz et al. 2020; Garcia et al. 2019; 2021; Silva et al. 2023; Campo et al. 2025). To date, no herrerasaurid remains have been described from strata assigned to the *Exaeretodon* Biozone of the Ischigualasto Formation, which overlies the *Scaphonyx*-*Exaeretodon*-*Herrerasaurus* Biozone (Martínez et al. 2013). These younger strata are characterized by an increase in the abundance of the traversodontid cynodont *Exaeretodon* relative to that of rhynchosaurs. In southern Brazil, the stratigraphic framework of the *Hyperodapedon* AZ is less well resolved because of the discontinuous nature of the exposed outcrops (Da-Rosa and Faccini 2005). Nevertheless, several fossiliferous localities have yielded assemblages dominated by the traversodontid cynodont *Exaeretodon*, typically accompanied by a marked decline in the abundance of rhynchosaurs (Langer et al. 2007). Consequently, the *Hyperodapedon* AZ has been subdivided into the *Hyperodapedon* Acme Zone (*sensu* Langer et al. 2007) at its base and the *Exaeretodon* sub-AZ (*sensu* Müller and Garcia 2020b) at its top. Similar to the apparently coeval deposits of Argentina, no herrerasaurid dinosaurs have so far been reported from Brazilian outcrops assigned to the *Exaeretodon* sub-AZ (Müller In press).

The absence of herrerasaurid records from strata characterized by abundance of *Exaeretodon* could reflect faunal changes that ultimately prevented the persistence of the group. For example, if herrerasaurids were ecologically specialized for preying upon rhynchosaurs, the decline of these herbivores may have negatively affected their survival. However, although rhynchosaurs become less abundant in these younger assemblages, they are not absent from the fauna (Langer et al. 2007; Schiefelbein et al. 2026). Consequently, this alone may not adequately explain the apparent absence of herrerasaurids from these deposits. Here, I describe the first herrerasaurid dinosaur from a fossil locality typically assigned to the *Exaeretodon* sub-AZ of southern Brazil. This discovery demonstrates that herrerasaurids persisted in the region despite the major faunal turnover associated with the transition from rhynchosaur-dominated to *Exaeretodon*-dominated assemblages, extending the known temporal range of the clade.

### Institutional abbreviations

AMNH, American Museum of Natural History, New York, U.S.A.; CAPPA/UFSM, Centro de Apoio à Pesquisa Paleontológica da Quarta Colônia da Universidade Federal de Santa Maria, São João do Polêsine, Brazil; GR, Ghost Ranch Ruth Hall Museum of Paleontology, Ghost Ranch, New Mexico, U.S.A.; ISI, Indian Statistical Institute, Kolkata, India; MBM, Mount Blanco Museum, Crosbyton, Texas, U.S.A.; MCZ, Museum of Comparative Zoology, Cambridge; PVL, Paleontología de Vertebrados, Instituto “Miguel Lillo”, San Miguel de Tucumán, Argentina; MSM, Mesa Southwest Museum, Mesa, Arizona; U.S.A.; NHMZ, Natural History Museum of Zimbabwe, Bulawayo, Zimbabwe; PVSJ, Division of Vertebrate Paleontology of the Museo de Ciencias Naturales de la Universidade Nacional de San Juan, San Juan, Argentina; SMNS, Staatliches Museum für Naturkunde, Stuttgart, Germany; TMM, Vertebrate Paleontology Laboratory, University of Texas at Austin, Austin, U.S.A.; TTU, Texas Tech University Museum, Lubbock, Texas, U.S.A.; UCMP, University of California, Berkeley, California; UFSM, Laboratório de Estratigrafia e Paleobiologia, Universidade Federal de Santa Maria, Santa Maria, Brazil; ULBRA, Centro de Apoio à Pesquisa Paleontológica da Quarta Colônia, Universidade Federal de Santa Maria, São João do Polêsine, Brazil (previously Museu de Ciências Naturais, Universidade Luterana do Brasil, Canoas, Rio Grande do Sul, Brazil)

### Geological setting

**Fig. 1:**
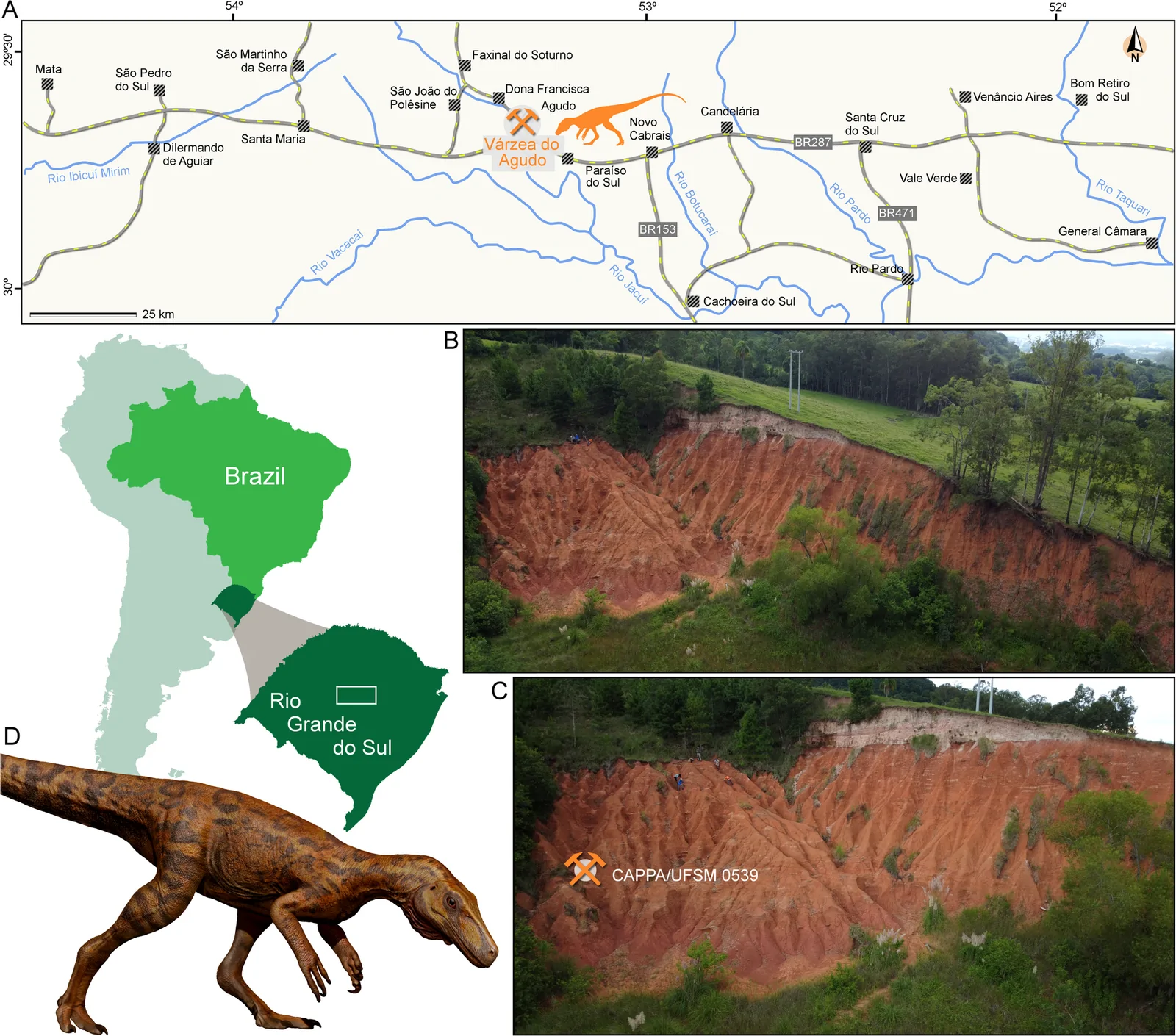
Study area and provenance of CAPPA/UFSM 0539. **A** Map of the central region of Rio Grande do Sul, Brazil, indicating the location of the Várzea do Agudo (= Janner) site. **B** General view of the Várzea do Agudo site. **C** Overview of the Várzea do Agudo site showing the location where CAPPA/UFSM 0539 was collected *ex situ*. **D** Artistic representation of CAPPA/UFSM 0539 in life (by Johnny Pauly V. Mingau)

The specimen was collected from the Várzea do Agudo site (= Janner site), located in the municipality of Agudo (29°39′10.89″S, 53°17′34.20″W), Rio Grande do Sul, Brazil (Fig. 1). The fossil-bearing strata at this site belong to the Santa Maria Formation (Paraná Basin) and are assigned to the Candelária Sequence of the Santa Maria Supersequence (Zerfass et al. 2003; Horn et al. 2014). The fossil assemblage from the Várzea do Agudo site is of Carnian age (Langer et al. 2007), although the uppermost fossil-bearing beds may extend into the earliest Norian (Roese-Miron et al. 2025). The age of the site is currently inferred based on biostratigraphic evidence. The co-occurrence of the rhynchosaur genus *Hyperodapedon* and the traversodontid cynodont *Exaeretodon* is characteristic of the *Hyperodapedon* AZ (Langer et al. 2007). However, the fossil-bearing succession at the Várzea do Agudo site extends over approximately 10 m of stratigraphic thickness, and the traversodontid cynodont *Siriusgnathus* has been recorded exclusively in the uppermost portion of the section (Roese-Miron et al. 2025). Notably, *Exaeretodon* has not yet been recovered from this same stratigraphic level. This distribution suggests that the exposed succession at the Várzea do Agudo site may encompass two distinct AZs: a lower interval corresponding to the *Hyperodapedon* AZ, characterized by abundant *Exaeretodon* remains (Müller et al. 2015) and occasional rhynchosaur fossils, and an upper, younger interval that is, so far, characterized exclusively by *Siriusgnathus*. Although strata assigned to the *Hyperodapedon* AZ have been radiometrically dated at another Brazilian fossil locality, indicating a Carnian age (233.23 ± 0.73; Langer et al. 2018), the *Siriusgnathus*-bearing beds have not yet been directly dated. Based on biostratigraphic investigations, these strata have been suggested to be of latest Carnian or earliest Norian age (Pavanatto et al. 2018; Roese-Miron et al. 2020; Müller 2021; Doering et al. 2024; Dalle-Laste et al. 2025).

The exposed strata at the Várzea do Agudo site comprise a stratigraphic succession approximately 12 to 13 m thick (Fig. 1B). Fossils are restricted to the lower 10 m of the section, occurring both in the lowermost portion, which consists of approximately 3 m of red mudstones, and in the overlying portion, composed of approximately 7 m of orange mudstones. The lower red mudstone portion has yielded only the hyperodapedontine rhynchosaur *Isodapedon varzealis* (Schiefelbein et al. 2026). In contrast, the overlying orange mudstones have produced rhynchosaurs of the genus *Hyperodapedon* (Langer et al. 2007), the traversodontid cynodont *Exaeretodon riograndensis* (Oliveira et al. 2007; Kerber et al. 2025a, b a), the ecteniniid cynodont *Trucidocynodon riograndensis* (Oliveira et al. 2010; Stefanello et al. 2018; Kerber et al. 2025a, b b), the proterochampsian archosauriform *Stenoscelida aurantiacus* (Müller et al. 2022), the ornithosuchid pseudosuchian *Dynamosuchus collisensis* (Müller et al. 2020), and the sauropodomorph dinosaurs *Pampadromaeus barberenai* (Cabreira et al. 2011) and *Bagualosaurus agudoensis* (Pretto et al. 2019). In addition, as noted above, the uppermost levels of the orange mudstones have yielded the traversodontid cynodont *Siriusgnathus niemeyerorum*, which is currently the only formally recognized taxon from this portion (Roese-Miron et al. 2025).

The specimen described here was recovered from the orange mudstones (Fig. 1C), specifically from the portion characterized by the abundance of *Exaeretodon*. The exact position of the material within this portion of the outcrop is unclear because it was not found in situ. Several authors have interpreted this portion of the outcrop as overlying the *Hyperodapedon* Acme Zone (Langer et al. 2007) and have referred to it as the *Exaeretodon* sub-AZ (Müller and Garcia 2020b). Accordingly, within this biostratigraphic framework, the new specimen is assigned to the *Exaeretodon* sub-AZ and is considered to be Carnian in age.

### Materials and methods

The material described here is housed in the palaeontological collection of the Centro de Apoio à Pesquisa Paleontológica da Quarta Colônia, Universidade Federal de Santa Maria (CAPPA/UFSM), located in the municipality of São João do Polêsine, Rio Grande do Sul, Brazil. The specimen, catalogued as CAPPA/UFSM 0539, consists of the distal portion of a left pubis. The fossil was originally encased in a carbonate concretion, which was removed during mechanical preparation using pneumatic air scribes. A three-dimensional model of CAPPA/UFSM 0539 was generated by digitally scanning the specimen using Abound v3.1.0, producing a mesh comprising fewer than 30 million triangles and an 8 K texture. The resulting model was subsequently processed in MeshLab v.2023.12. The final 3D model of CAPPA/UFSM 0539 is available at: https://figshare.com/s/5690432ab98a44e46380.

Although CAPPA/UFSM 0539 is represented by an extremely fragmentary specimen, it consists of a bone element that bears specific features found in only a few archosaur groups. Therefore, it can be expected that a more resolved phylogenetic placement within a less inclusive clade may still be possible. To investigate the phylogenetic affinities of this material and assess its taxonomic assignment, the specimen was included in a phylogenetic analysis. For this purpose, CAPPA/UFSM 0539 was scored in the matrix that combines the matrices of Ezcurra et al. (2025) and Müller (2025), both of which includes a broad diversity of avemetatarsalian archosaurs, with emphasis on Triassic forms. *Soumyasaurus aenigmaticus* was excluded from the data matrix due to its fragmentary nature and taxonomic issues (Garcia and Müller 2025; Paes-Neto et al. 2025). Similarly, *Nhandumirim waldsangae* was removed because it has been considered a possible junior synonym of *Saturnalia tupiniquim*, and the material used to score this operational taxonomic unit (OTU) represents a skeletally immature individual (Fonseca et al. 2026; Srivastava and Nesbitt 2026). Some pubic character scores were updated based on a re-examination of the OTUs and a new character on the anterior expansion of the distal end of the pubis was included (character 293). A complete list of these changes is provided in the Supplementary Material. The updated data matrix comprises 293 morphological characters and 79 OTUs. The data matrix was analyzed in TNT v. 1.6 (Goloboff and Morales 2023) to recover the most parsimonious trees (MPTs). All characters were equally weighted, and characters 4, 13, 18, 25, 63, 82, 84, 87, 89, 109, 142, 166, 174, 175, 184, 186, 190, 201, 203, 205, 209, 212, 225, 235, 236, 239, 250, 256, and 291 were treated as ordered, consistent with previous iterations of this data matrix. *Euparkeria capensis* was used to root the MPTs, which were recovered using the random addition sequence + tree bisection reconnection (TBR), which included 1000 replicates of Wagner trees (with random seed = 0), TBR and branch-swapping (holding 20 trees saved per replicate). Nodes without synapomorphies were collapsed after the search. Topologies retained as replicates were branch-swapped for MPTs using TBR. The strict consensus tree was constructed using all trees recovered in the analysis and included all OTUs. The consistency index (CI) and retention index (RI) were calculated using the script STATSb.run developed by Spiekman et al. (2021). The data matrix, list of morphological characters, and list of modifications are available at: https://figshare.com/s/5690432ab98a44e46380.

### Systematic paleontology

DINOSAURIA Owen 1842

SAURISCHIA Seeley 1887

HERRERASAURIDAE Benedetto 1973

#### Material

**Fig. 2:**
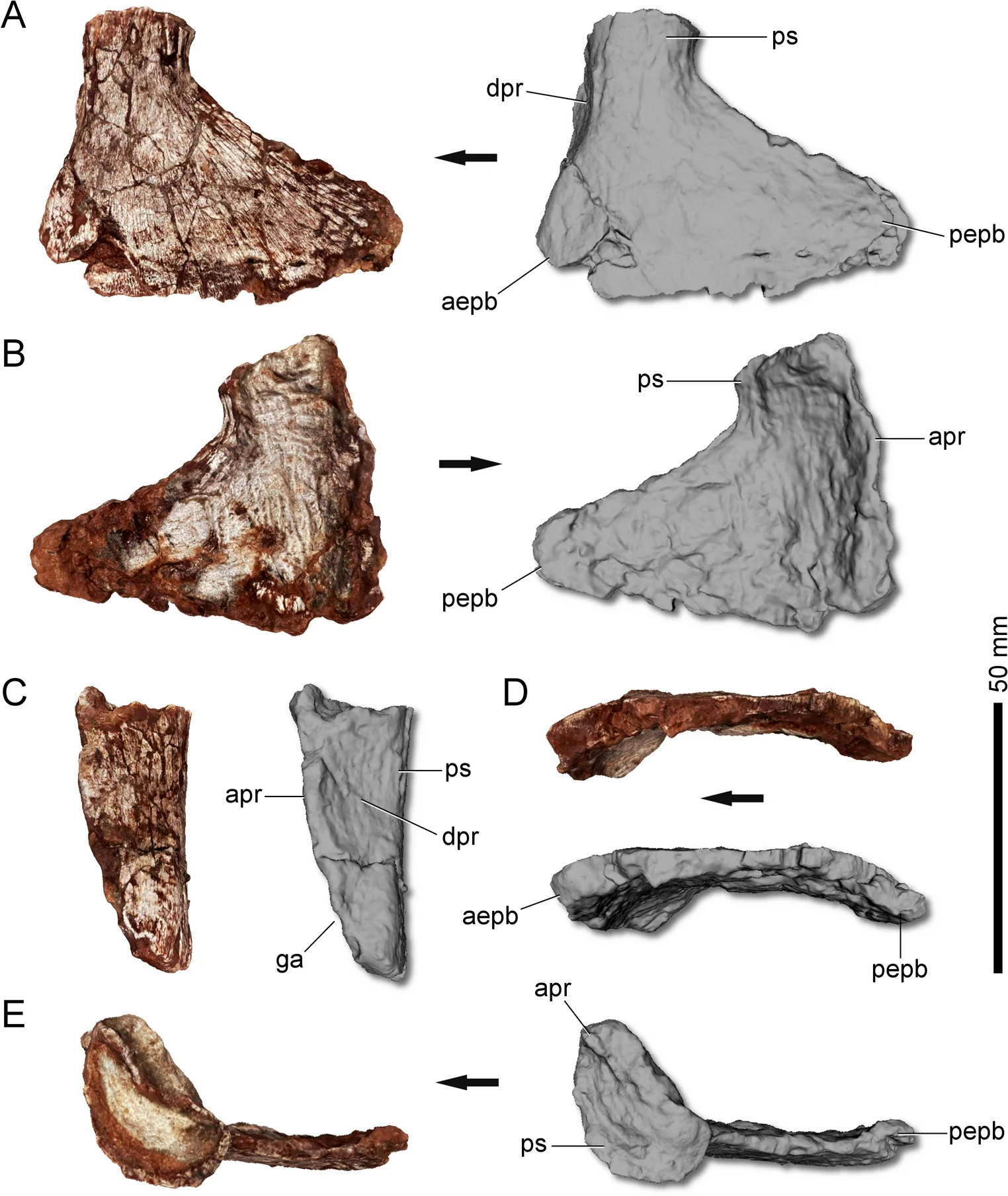
Left pubis of CAPPA/UFSM 0539 in lateral (**A**), medial (**B**), anterior (**C**), ventral (**D**), and (**E**) dorsal views. The black arrows indicate the anterior direction. Abbreviations: aepb, anterior extension of the pubic boot; apr, apron; dpr, depression; pepb, posterior extension of the pubic boot; ps, pubic shaft; ga, gap

CAPPA/UFSM 0539. Distal portion of a left pubis (Fig. 2)

#### Locality, age, and horizon

Várzea do Agudo site (= Janner site) is in the municipality of Agudo (29°39′10.89″S, 53°17′34.20″W), Rio Grande do Sul, Brazil (Fig. 1). The fossil-bearing strata at the site belong to the Candelária Sequence of the Santa Maria Supersequence (Zerfass et al. 2003; Langer et al. 2007; Horn et al. 2014). Its fossil assemblage is assigned to the Exaeretodon sub-AZ of the Hyperodapedon AZ (Langer et al. 2007; Müller & Garcia 2020b). The Hyperodapedon AZ has been dated to the Carnian based on radioisotopic data (Langer et al. 2018).

#### Description

The specimen preserves the complete distal end of a left pubis (Fig. 2). The lateral surface is fully exposed, whereas some matrix was intentionally left on the medial surface to avoid further weakening the element, which is exceptionally thin. The bone surface is relatively well preserved, with a series of rugose structures along the lateral margin of the ventralmost portion (Fig. 2A). The pubic shaft is kidney-shaped in cross-section at its proximalmost preserved portion (Fig. 2E). The bone wall is extremely thin, measuring 2.5 mm at its thickest point and 1.2 mm at its thinnest. As a result, the medullary cavity is proportionally wide. At the proximalmost preserved portion (i.e., the unexpanded region), the shaft measures 19 mm in anteroposterior length and 21.4 mm in transverse width. In cross-section, the shaft is laterally convex and tapers medially to form the pubic apron, which is anteroposteriorly thin and forms a plate-like structure (Fig. 2B)

Ventral to the proximalmost preserved portion of the shaft, the bone begins to project posteriorly, forming a remarkably elongate, triangular process (i.e., the pubic boot) in lateral view (Fig. 2A), such as in other herrerasaurids. This well-developed expansion differs from the poorly expanded, knob-like distal pubis observed in several Carnian saurischians, such as *Buriolestes schultzi* (ULBRA-PVT280; Cabreira et al. 2016), *Mbiresaurus raathi* (NHMZ 2547; Griffin et al. 2022), *Eodromaeus murphi* (PVSJ 562; Martínez et al. 2011), and *Anteavis crurilongus* (PVSJ 1085; Martínez et al. 2025). Among non-herrerasaurid herrerasaurians, the distal end of the pubis is strongly posteriorly expanded in *Tawa hallae* (GR 155; Nesbitt et al. 2009), resembling the condition in CAPPA/UFSM 0539, whereas the posterior expansion is much weaker in *Maleriraptor kuttyi* (ISIR 282; Ezcurra et al. 2025), contrasting with the condition observed in herrerasaurids. The transition from the pubic shaft to the dorsal surface of the pubic boot is gradual, and the main axis of the boot forms an angle approaching 90° with the long axis of the shaft. This condition differs from the upturned posterior expansion of the pubic boot observed in some poposauroids, such as *Shuvosaurus inexpectatus* (TTU-P9001; Nesbitt and Chatterjee 2024), in which the posterior expansion is dorsally deflected, producing a hook-like structure and forming a more acute angle with the pubic shaft. The pubic boot of CAPPA/UFSM 0539 is not restricted to a posterior expansion, but also presents a subtle anterior expansion that diverges from the pubic shaft more ventrally than the posterior expansion (Fig. 2A). This anterior expansion is considerably smaller and projects from the shaft at an even more gradual angle than the posterior expansion. Among herrerasaurians, the anterior expansion is absent in *Staurikosaurus price*i (MCZ 1669; Bittencourt and Kellner 2009) and *Tawa hallae* (GR 155; Nesbitt et al. 2009). In contrast, this structure is present and well-developed in *Gnathovorax cabreirai* (CAPPA/UFSM 0009; Pacheco et al. 2019), *Sanjuansaurus gordilloi* (PVSJ 605; Alcober and Martínez 2010), and *Herrerasaurus ischigualastensis* (PVL 2566; Novas 1994). Medially, at the level where the anterior expansion begins, a shallow depression extends anteriorly between the pubic apron and the pubic shaft (Fig. 2C). The total length of the pubic boot, including both the anterior and posterior expansions, is 69.6 mm. At its ventralmost portion, the pubic boot is transversely only 3 mm thick. This extreme thinness results from the absence of a medial expansion of the distal pubis, leaving the distal end L-shaped in ventral view (Fig. 2D). This morphology resembles that of other herrerasaurids, whereas in theropods such as *Dilophosaurus wetherilli* (UCMP 37302; Marsh and Rowe 2020), the distal end is transversely broader.

The pubic apron merges with the pubic shaft before reaching the distal end of the pubis. As a result, a ventral gap (Fig. 2C) was present between the paired pubic aprons at their distal ends. A similar gap between the conjoined pubes is also present in other herrerasaurids and early saurischians, such as *Buriolestes schultzi* (ULBRA-PVT280; Cabreira et al. 2016), *Eodromaeus murphi* (PVSJ 562; Martínez et al. 2011), and *Maleriraptor kuttyi* (ISIR 282; Ezcurra et al. 2025). Similarly, complete contact along the entire ventral margin of the pubic boot was likely absent because, as noted above, the distal end lacks a medial expansion. Nevertheless, the medial margin of the pubic boot is gently concave in ventral view, suggesting that the posteriormost portion of the pubic boot may still have contacted its counterpart (Fig. 2D).

## Results

**Fig. 3:**
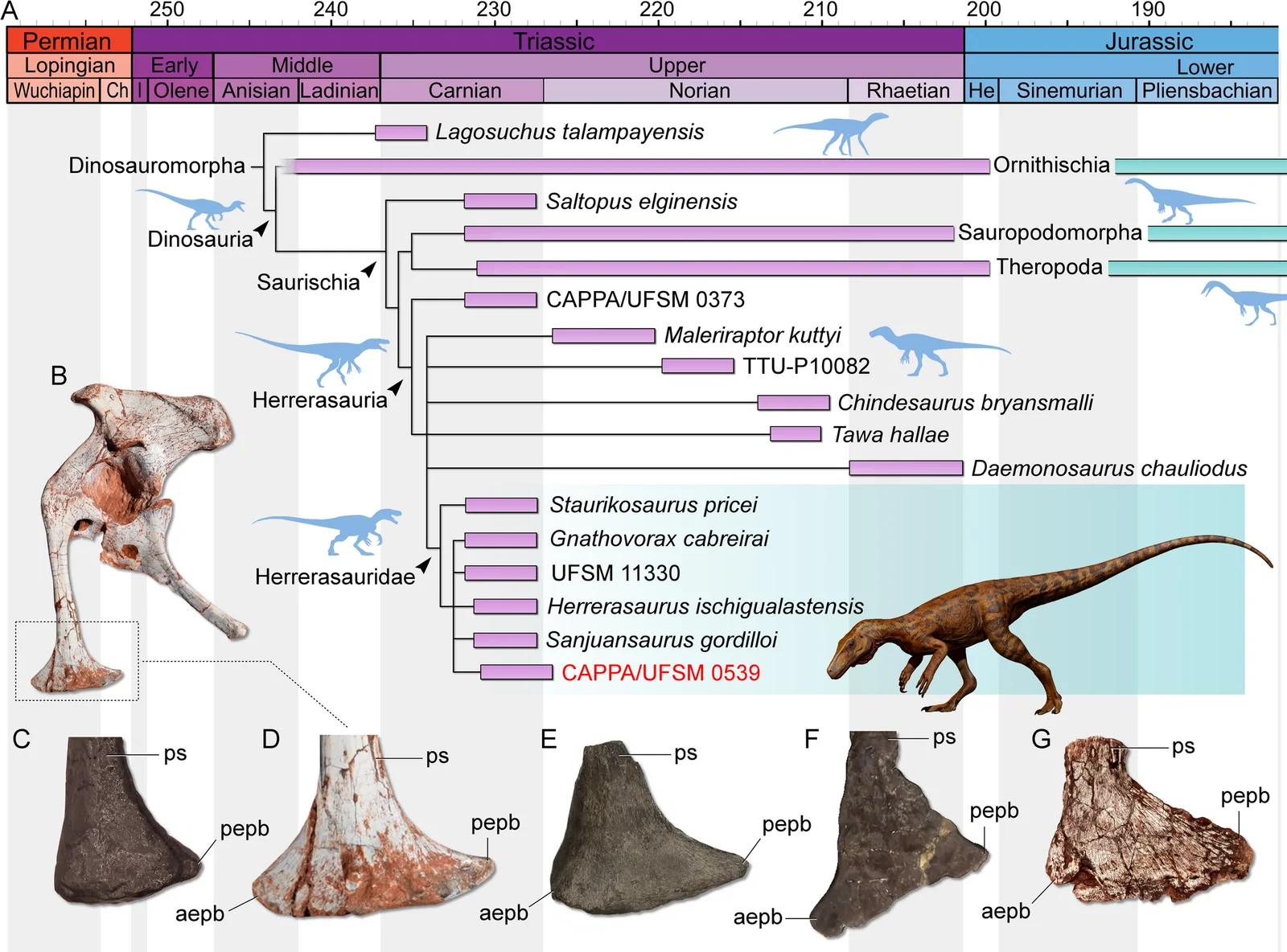
Phylogenetic analysis results and morphological variation of the herrerasaurid pubic boot. **A** Time-calibrated reduced strict consensus tree depicting the phylogenetic position of CAPPA/UFSM 0539. Life reconstruction of CAPPA/UFSM 0539 by Johnny Pauly V. (Mingau). Silhouettes based on the artwork of Matheus F. Gadelha, Marcio L. Castro, and Caio Fantini. **B** Right pelvic girdle of *Gnathovorax cabreirai* (CAPPA/UFSM 0009) in lateral view (reversed). Distal end of the pubis of **C** a cast of *Staurikosaurus pricei* (MCZ 1669; right element, reversed), **D***Gnathovorax cabreirai* (CAPPA/UFSM 0009; right element, reversed), **E***Herrerasaurus ischigualastensis* (PVSJ 373; left element), **F***Sanjuansaurus gordilloi* (PVSJ 605; right element, reversed), and **G** CAPPA/UFSM 0539 (left element), in lateral view. Elements are not to scale. Abbreviations: aepb, anterior extension of the pubic boot; pepb, posterior extension of the pubic boot; ps, pubic shaft

The phylogenetic analysis recovered 540 MPTs with 1131 steps (CI = 0.29339; RI = 0.68951). The new specimen is recovered within Herrerasauridae in all MPTs. In the strict consensus tree (Fig. 3A), CAPPA/UFSM 0539 forms a polytomy with UFSM 11,330 (an indeterminate herrerasaurid from Brazil; Garcia et al. 2021), *Gnathovorax cabreirai*, *Herrerasaurus ischigualastensis*, and *Sanjuansaurus gordilloi*. This clade is supported by five synapomorphies, only one of which is related to the pubis: an anteriorly expanded distal end of the pubis (character 293: 0→1; Fig. 3D–G). The polytomy is recovered as the sister group to *Staurikosaurus pricei*, the earliest-diverging taxon of Herrerasauridae. This relationship is supported by eight synapomorphies, of which only one involves the pubis: the distal end of the pubis is beveled (character 191: 0→1). Herrerasauridae is recovered within Herrerasauria, which also includes taxa from the Triassic of the United States, India, and Brazil. Herrerasauria is recovered as the sister group to the clade comprising Theropoda + Sauropodomorpha (i.e., Eusaurischia). *Saltopus elginensis* is recovered as the earliest-diverging saurischian. As in previous iterations of this data matrix, Saurischia forms the sister group to Ornithischia, the latter also including silesaurids as early-diverging members (e.g.Müller and Garcia 2020a; Norman et al. 2022; Ezcurra et al. 2025; Müller 2025). The Bremer support was 1, and bootstrap values (1,000 replicates) were below 50% for the nodes of interest. These low values are not surprising given the fragmentary nature of the material investigated, as well as that of other specimens included in the data matrix.

## Discussion

**Fig. 4:**
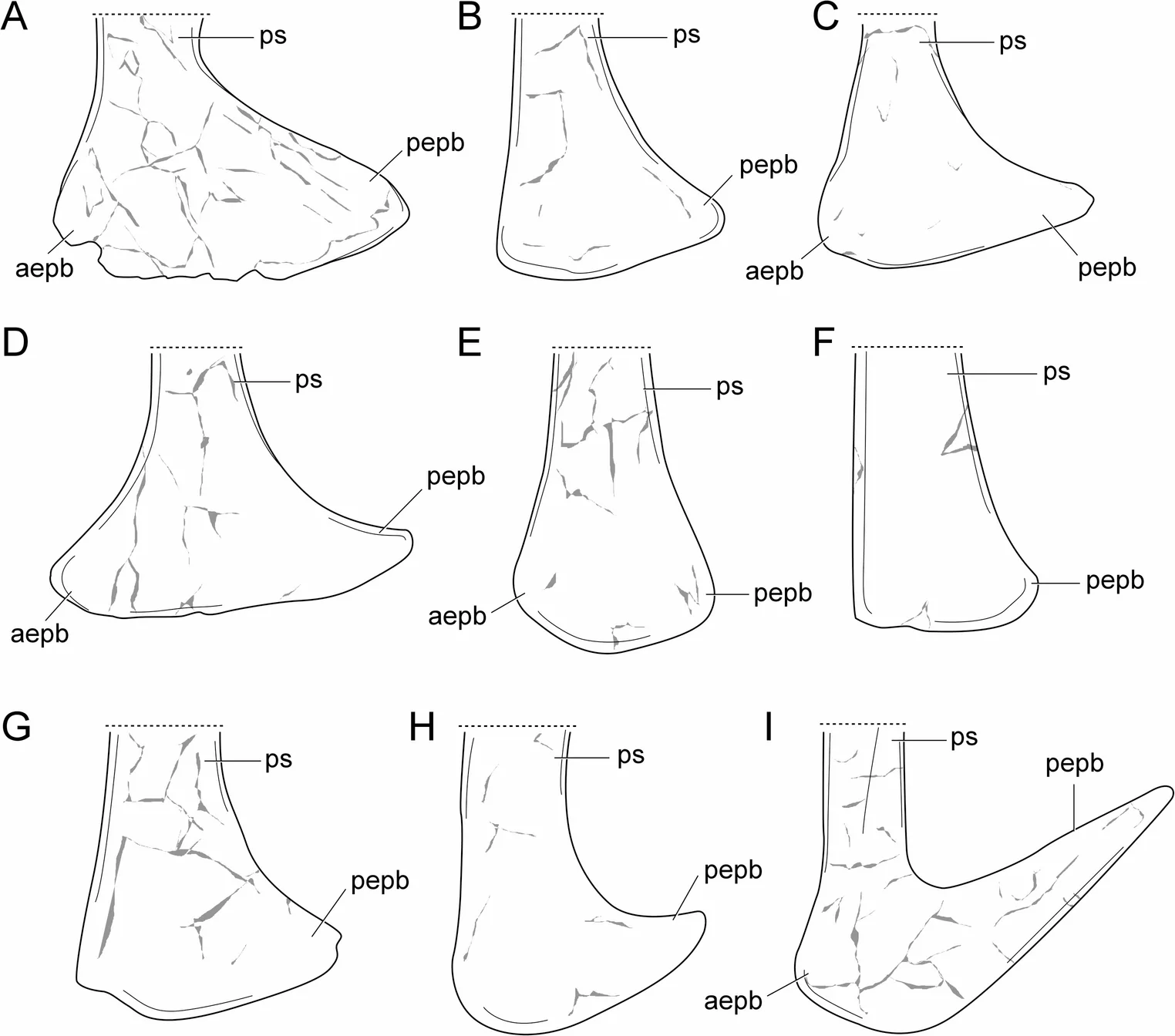
Interpretative drawings of the distal end of the pubis of selected archosaurs in lateral view. **A** CAPPA/UFSM 0539. **B** Cast of *Staurikosaurus pricei* (MCZ 1669; right element, reversed). **C ***Herrerasaurus ischigualastensis* (PVSJ 373; left element). **D ***Gnathovorax cabreirai* (CAPPA/UFSM 0009; right element, reversed). **E ***Dilophosaurus wetherilli* (UCMP 37302; right element, reversed). **F ***Arizonasaurus babbitti* (MSM 4590; right element, reversed). **G ***Batrachotomus kupferzellensis* (SMNS 80270; left element). **H ***Postosuchus kirkpatricki* (‘MBM’ specimen). **I ***Shuvosaurus inexpectatus* (TTU-P9001). Elements are not to scale. Abbreviations: aepb, anterior extension of the pubic boot; pepb, posterior extension of the pubic boot; ps, pubic shaft

Despite the fragmentary nature of CAPPA/UFSM 0539, the preserved portion of the specimen allows its confident referral to Herrerasauridae, as it possesses a combination of morphological features currently known to be exclusive to members of this clade. Whereas a small distal expansion of the pubis (i.e., a knob) is present in some members of several archosaur clades (e.g., crocodylomorphs, theropods, sauropodomorphs), a well-expanded distal pubic end (i.e., pubic boot) is restricted among Triassic archosaurs to some pseudosuchians, theropods, and herrerasaurids (Nesbitt 2011; Ezcurra et al. 2025). However, the morphology of the pubic boot differs among these groups. Among pseudosuchians, this condition is present in some suchians (Fig. 4), such as *Batrachotomus kupferzellensis* (SMNS 80270; Gower and Schoch 2009); *Postosuchus kirkpatricki* (‘MBM’ specimen; Weinbaum 2013), *Arizonasaurus babbitti* (MSM 4590; Nesbitt 2005), and *Effigia okeeffeae* AMNH FR 30587; Nesbitt 2007), and *Shuvosaurus inexpectatus* (TTU-P9001; Nesbitt and Chatterjee 2024). In non-poposauroid suchians, the distal expansion of the pubis is mediolaterally thick, and the pubic expansions meet at the midline (Nesbitt 2011). This morphology clearly distinguishes these taxa from CAPPA/UFSM 0539, in which the distal expansion is transversely thin. In contrast, some poposauroids possess a transversely flattened distal expansion that is restricted to the lateral margin of the pubis, resulting in distal expansions that remain well separated from each other. This condition typically produces an L-shaped pubis in ventral view (Nesbitt 2011), as also observed in CAPPA/UFSM 0539. However, in poposauroids possessing this morphology, the posterior expansion is hook-shaped in lateral view, with its posterior margin directed dorsally. This differs from the condition in CAPPA/UFSM 0539, in which the posterior expansion does not project dorsally. In Triassic theropods that possess a pubic boot, such as *Coelophysis bauri* (AMNH FR 7224; Nesbitt 2011), the posterior expansion is far less developed than in CAPPA/UFSM 0539. Even in Early Jurassic theropods, such as *Dilophosaurus wetherilli* (UCMP 37302; TMM 43646-1; Marsh and Rowe 2020), the posterior expansion (Fig. 4E) remains considerably less developed than in CAPPA/UFSM 0539. The pubic boot of CAPPA/UFSM 0539 also differs from those of these theropods in the morphology of its distal expansion, which results from the lateral portion of the distal pubis being deflected posteriorly. Consequently, the lateral margin of the distal pubis lies posterior to the medial margin, whereas in these theropods both margins lie at the same anteroposterior level (Langer and Benton 2006). Finally, CAPPA/UFSM 0539 differs from non-herrerasaurid herrerasaurians (e.g., *Tawa hallae* and TTU P10082; Nesbitt et al. 2009; Sarıgül 2017) and *Staurikosaurus pricei* (Fig. 3C) in possessing an anterior expansion of the distal end of the pubis. In these herrerasaurians, the distal expansion is directed exclusively posteriorly. Thus, the combination of pubic characters present in CAPPA/UFSM 0539 is known only among some members of Herrerasauridae within Triassic archosaurs (Fig. 3D–F). This interpretation is further supported by the phylogenetic analysis conducted in the present study, which, despite being limited by the fragmentary nature of the new specimen, nevertheless recovered CAPPA/UFSM 0539 in a relatively less inclusive position within Herrerasauridae. Although the new specimen differs from *Staurikosaurus pricei* in possessing an anterior expansion of the distal end of the pubis, which is absent in *Staurikosaurus pricei*, it cannot be confidently referred to any of the remaining species. CAPPA/UFSM 0539 lacks features that are uniquely shared with any of the other three recognized herrerasaurid species. Accordingly, the referral of CAPPA/UFSM 0539 to an indeterminate herrerasaurid appears to be the most plausible hypothesis, and no alternative interpretation is currently supported by comparable evidence. A possible alternative scenario would place CAPPA/UFSM 0539 elsewhere within Herrerasauria if future discoveries demonstrate that the anterior expansion of the distal pubis was more widely distributed across the clade than is currently recognized. However, based on the material presently available, this hypothesis remains less plausible.

Considering the growing number of new herrerasaurid specimens reported from the Triassic beds of Brazil (Garcia et al. 2021; Silva et al. 2023; Campo et al. 2025), the discovery of a new representative from the *Hyperodapedon* AZ is not unexpected. However, despite this increase in the known diversity of the group, no record of Herrerasauridae had previously been documented from the *Exaeretodon* sub-AZ  (Müller in press). CAPPA/UFSM 0539 demonstrates that herrerasaurid dinosaurs persisted as components of these ecosystems even in deposits characterized by the decline of rhynchosaurs and the increasing abundance of traversodontid cynodonts. This occurrence may suggest that the clade possessed a certain degree of ecological plasticity, allowing its members to persist in ecosystems that differed from those typically associated with the *Hyperodapedon* Acme Zone. The identity of the prey exploited by herrerasaurids in these ecosystems remains difficult to determine because of the lack of direct evidence of predator-prey interactions. Nevertheless, the discovery of CAPPA/UFSM 0539 increases the diversity of relatively large-bodied predators known from the Várzea do Agudo site, whose faunal assemblage also includes proterochampsians, ornithosuchids, ecteniniids, and early sauropodomorphs interpreted as having incorporated vertebrate prey into their diet (Oliveira et al. 2010; Cabreira et al. 2011; Müller et al. 2020, 2022; Kerber et al. 2025a, b b). Based on more complete herrerasaurid specimens from other Brazilian (Garcia et al. 2021) and Argentinean localities (Novas 1986), members of the clade were capable of attaining relatively large body sizes. Consequently, among the carnivorous taxa recorded at the Várzea do Agudo site, herrerasaurids were likely the largest predators when fully grown. Accordingly, CAPPA/UFSM 0539 not only expands the diversity of predators known from the site but also suggests that herrerasaurid dinosaurs may have occupied the role of apex predators within the food webs of these ecosystems.

**Fig. 5:**
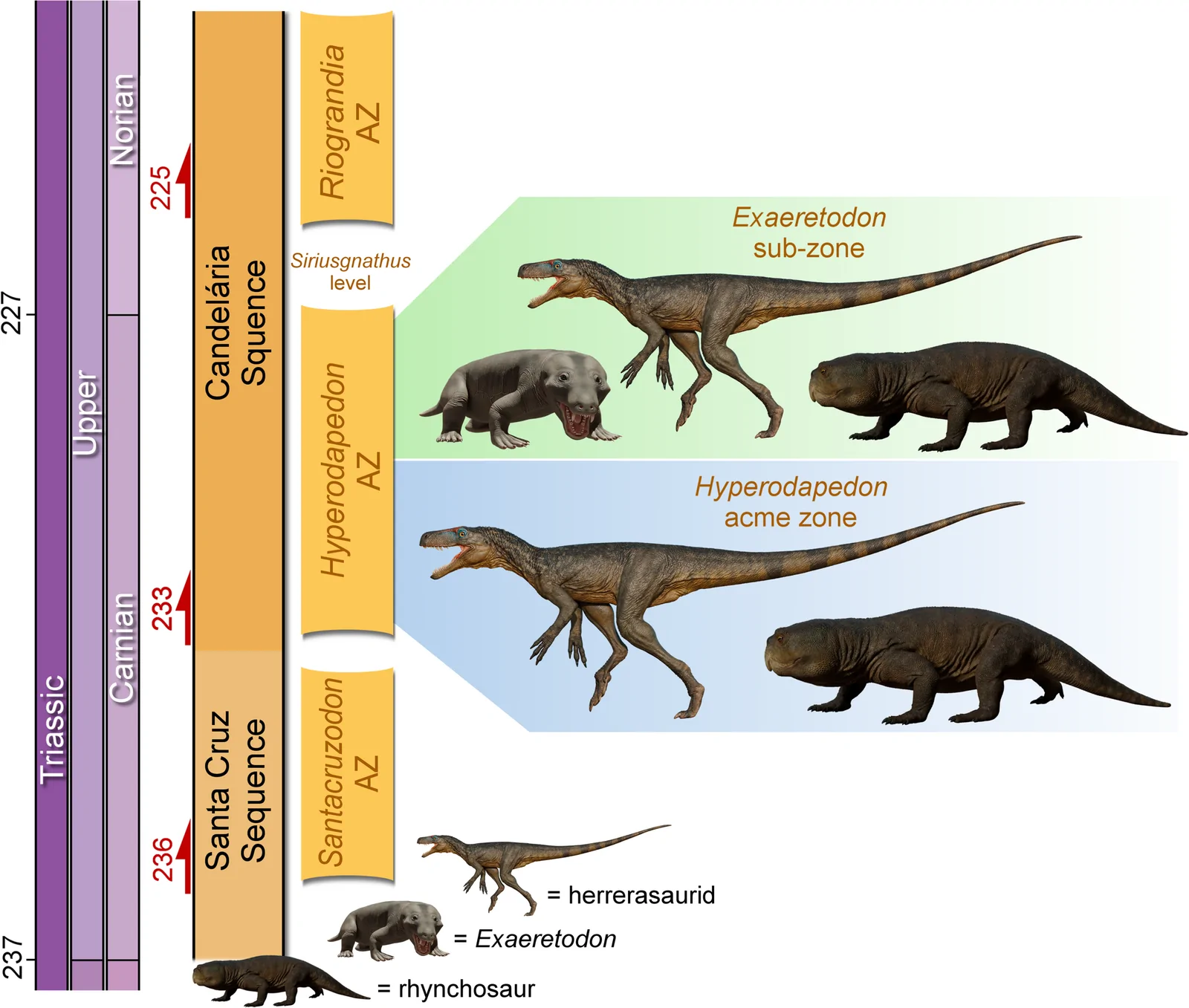
Stratigraphic distribution of herrerasaurid dinosaurs in the Late Triassic of southern Brazil in light of the discovery of CAPPA/UFSM 0539. Life reconstructions of rhynchosaurs and herrerasaurids by Caio Fantini, and of *Exaeretodon* by Márcio L. Castro

In addition to increasing the diversity of taxa recorded from the *Exaeretodon* sub-zone, CAPPA/UFSM 0539 may also represent the youngest known record of Herrerasauridae worldwide (Fig. 5). In the stratigraphic succession of the Ischigualasto Formation, Argentina, the *Exaeretodon* biozone is unequivocally younger than the *Scaphonyx*-*Exaeretodon*-*Herrerasaurus* biozone, as the strata assigned to the *Exaeretodon* biozone overlap those of the latter biozone (Martínez et al. 2013). If the Brazilian strata characterized by the abundance of *Exaeretodon* follow the same pattern, CAPPA/UFSM 0539 would be younger than *Gnathovorax cabreirai*, *Staurikosaurus pricei*, and the indeterminate herrerasaurid remains reported by Garcia et al. (2021) and Silva et al. (2023). Furthermore, the closest relatives known from younger strata elsewhere in the world are non-herrerasaurid herrerasaurians (Novas et al. 2021; Garcia et al. 2024; Ezcurra et al. 2025; Srivastava and Nesbitt 2026). Nevertheless, absolute dating of the Brazilian strata remains essential to establish a more reliable chronological framework for the different Triassic fossil-bearing localities.

## Conclusions

The new specimen preserves a combination of pubic features shared exclusively with members of Herrerasauridae. Its referral to an indeterminate herrerasaurid is supported by both morphological and phylogenetic evidence. Accordingly, CAPPA/UFSM 0539 represents the first record of a herrerasaurid dinosaur from the *Exaeretodon*-dominated strata of the *Hyperodapedon* AZ. This finding indicates that herrerasaurids persisted in ecosystems distinct from those typical of the *Hyperodapedon* Acme Zone. Furthermore, CAPPA/UFSM 0539 may represent the youngest known record of Herrerasauridae worldwide.

## Supplementary Information

Supplementary Material 1.

Supplementary Material 2.

Supplementary Material 3.

## Data Availability

The data matrix and character list used in the phylogenetic analysis, as well as the 3D models of the new specimen, are available as supplementary material and at the following link: https://figshare.com/s/5690432ab98a44e46380.
