# Supplementary material for: First occurrence of a herrerasaurid dinosaur in a traversodontid cynodont-dominated Late Triassic fauna of southern Brazil
Source: Naturwissenschaften. 2026 Oct 19;113(6):133. doi: 10.1007/s00114-026-02185-1 (PMC13680303; doi:10.1007/s00114-026-02185-1)
Supplement: Supplementary file 1 [file 114_2026_2185_MOESM1_ESM.docx]

**SUPPLEMENTARY INFORMATION**

**First occurrence of a herrerasaurid dinosaur in a traversodontid cynodont-dominated Late Triassic fauna of southern Brazil**

Rodrigo T. Müller

Corresponding author email: rodrigotmuller@hotmail.com

**Outline of contents:**

1. List of morphological characters employed in the phylogenetic analysis

2. Modifications to previous versions of the phylogenetic data matrix

3. References for supplementary information

**1. List of morphological characters**

1. Skull; length (Gauthier 1986):

0, longer than two thirds of the femoral (or estimate of its) length;

1, shorter than, or subequal to, two thirds of the femoral (or estimate of its) length.

2. Skull; Narial fossa surrounding external nares on lateral surface of premaxilla, position of ventral margin of fossa relative to the ventral margin of the premaxilla (Butler *et al*. 2008):

0, closely approaches the ventral margin of the premaxilla;

1, separated by a broad flat margin from the ventral margin of the premaxilla.

3. Skull; External nares, position of the ventral margin (Butler *et al*. 2008; Baron *et al*. 2017):

0, below or level with the ventral margin of the orbits;

1, above the ventral margin of the orbits.

4. Skull; alveolar margin of the premaxilla-maxilla articulation (Gauthier 1986) ORDERED:

0, continuous/straight;

1, arched;

2, deeply arched (arch deeper than its distance to the external naris).

5. Skull; subnarial foramen (Benton & Clark 1988):

0, absent;

1, present.

6. Skull; External narial fenestra length (Yates 2003):

0, half or less than the orbit;

1, more than half of the orbit.

7. Skull; External narial fenestra; rostral edge (Yates 2007):

0, rostral or close to the midlength of the premaxillary body;

1, closer to the maxilla articulation than to the rostral edge of the premaxilla.

8. Skull; Antorbital fenestra; length (Langer 2004):

0, equal to or longer than the orbit;

1, shorter than the orbit.

9. Skull; Laterotemporal fenestra; rostral edge (Yates 2003):

0, caudal to the caudal edge of the orbit;

1, rostral to the caudal edge of the orbit.

10. Skull; Supratemporal fossa/fenestra (Gauthier 1986):

0, does no excavate the frontal bone;

1, excavates the frontal bone.

11. Skull; Posttemporal foramen/fossa, position (Butler *et al*. 2008):

0, totally enclosed with the paraoccipital process;

1, forms a notch or foramen in the dorsal margin of the paroccipital process, enclose dorsally by the squamosal.

12. Premaxilla; rostrodorsal process (Nesbitt 2011):

0, shorter than the craniocaudal length of the premaxillary body;

1, longer than, or subequal to, the craniocaudal length of the premaxillary body.

13. Premaxilla; caudodorsal process (Gauthier 1986) ORDERED:

0, extends caudally between nasal and maxilla;

1, restricted to the ventral-caudal margin of the external naris;

2, restricted to the ventral margin of the external naris.

14. Premaxilla, edentulous anterior region (Butler *et al*. 2008):

0, absent, first premaxillary tooth is positioned adjacent to the symphysis;

1, present, first premaxillary tooth is inset the width of one or more crowns.

15. Premaxilla, posterodorsal process (maxillary process, posterolateral process, subnarial process), length (Butler *et al*. 2008):

0, does not contact lacrimal;

1, contacts the lacrimal, excludes maxilla-nasal contact.

16. Premaxilla, posterodorsal process (maxillary process, posterolateral process, subnarial process), width (Baron *et al*. 2017):

0, wide, plate-like;

1, thin, bar like.

17. Premaxillary palate (Butler *et al*. 2008):

0, strongly arched, forming a deep, concave palate;

1, horizontal or only gently arched.

18. Premaxilla (caudodorsal process) nasal (cranioventral process) contact (Yates 2003) ORDERED:

0, broad sutured contact;

1, point contact;

2, no contact.

19. Premaxilla; narial fossa at the rostroventral corner of the naris (Sereno 1999):

0, absent or shallow;

1, deep.

20. Premaxillary foramen (anterior premaxillary foramen) (Yates 2007):

0, absent;

1, present.

21. Premaxilla-maxilla diastema (Butler *et al*. 2008):

0, absent, maxillary teeth continue to anterior end of maxilla;

1, present, substantial diastema of at least one crowns length between maxillary and premaxillary teeth.

22. Premaxilla; caudomedial process (Rauhut 2003):

0, absent;

1, present.

23. Maxilla; facial portion, rostral to the rostral edge of external antorbital fenestra (Nesbitt 2011):

0, shorter than caudal portion;

1, equal in length or longer than portion caudal to the rostral edge of fenestra.

24. Maxilla; buccal emargination separated from the ventral margin of the antorbital fossa (Butler 2005):

0, absent;

1, present.

25. Maxilla; rostrodorsal margin, except for the rostromedial process (Yates 2003) ORDERED:

0, straight;

1, slightly concave;

2, with a strong inflection at the base of the ascending ramus.

26. Maxilla; ventral margin of the antorbital fossa (Nesbitt 2011):

0, smooth (continuous to the more ventral area);

1, elevated relative to the ventral surface (sharp longitudinal ridge present).

27. Maxilla; antorbital fossa, rostrocaudal extension of its medial wall (Langer 2004):

0, extends onto the whole ventral border of the internal antorbital fenestra;

1, does not reach the caudoventral edge of the internal antorbital fenestra.

28. Maxilla; antorbital fossa, lateral surface of its medial wall, close to the base of the ascending process (Rauhut 2003):

0, smooth; or with subcircular or oval blind pockets;

1, with one or more foramen or fenestra sized perforations.

29. Maxilla; surface ventral to the external antorbital fenestra, except for the articulation area with the lacrimal (Nesbitt 2011):

0, significantly deeper (more than 50%) rostrally than caudally;

1, approximately the same depth throughout.

30. Nasal; caudolateral process (Yates 2003):

0, does not envelop part of the rostral ramus of the lacrimal;

1, envelops part of the rostral ramus of the lacrimal.

31. Nasal; contribution to the antorbital fossa (Sereno *et al*. 1994):

0, does not form part of the dorsal border of the antorbital fossa;

1, forms part of the dorsal border of the antorbital fossa.

32. Nasal; rostroventral process width (Yates 2007):

0, equally broad or narrower than the rostrodorsal process at the basal portion;

1, wider than the rostrodorsal process at the basal portion.

33. Lacrimal; shape (Sereno 1999):

0, does not fold over the caudodorsal part of the antorbital fenestra;

1, fold over the caudodorsal part of the antorbital fenestra.

34. Lacrimal; height (Rauhut, 2003):

0, significantly less than the height of the orbit and usually fails to reach the ventral margin of the orbit;

1, as high as the orbit and contacts the jugal at the level of the ventral margin of the orbit.

35. Lacrimal; dorsal exposure (Yates 2003):

0, exposed in dorsal view;

1, dorsal portion of the lacrimal does not reach the skull dorsal surface.

36. Prefrontal; bone sheet between the rostral and ventral processes (Müller *et al*. 2018):

0, absent;

1, present.

37. Postfrontal (Gauthier 1986):

0, present;

1, absent.

38. Squamosal; ventral process (Yates 2003):

0, wider than one quarter of its length;

1, narrower than one quarter of its length.

39. Squamosal; ventral process contribution to laterotemporal fenestra (Bittencourt *et al*. 2014):

0, more than half of the caudal margin of the laterotemporal fenestra;

1, less than half of the caudal margin of the laterotemporal fenestra.

40. Postorbital bar (Nesbitt 2011):

0, composed by both the jugal and postorbital in nearly equal proportions;

1, composed mostly by the postorbital.

41. Postorbital; rostral process (Ezcurra 2006):

0, equal to or longer than the caudal process;

1, shorter than the caudal process.

42. Jugal; long axis of the body (Nesbitt 2011):

0, nearly horizontal to the alveolar margin of the maxilla;

1, oblique to the alveolar margin of the maxilla.

43. Jugal; rostral and caudal rami ventral margin (Cabreira *et al*. 2016):

0, straight or forming an angle of more than 180°;

1, forming angle of less than 180°.

44. Jugal; rostral extent of the slot for the quadratojugal (Nesbitt 2011):

0, caudal to the caudal edge of the dorsal ramus of the jugal;

1, rostral to the caudal edge of the dorsal ramus of the jugal.

45. Jugal; rostral process (Rauhut, 2003):

0, participates in caudal edge of internal antorbital fenestra;

1, excluded from the internal antorbital fenestra by the lacrimal or maxilla.

46. Jugal; forked caudal process (Tykoski & Rowe 2004):

0, ventral tine longer than the dorsal one;

1, dorsal tine longer or subequal than the ventral one.

47. Jugal, caudal process; pecidel projecting the forking part of the bone caudally (Cabreira *et al*. 2016):

0, present;

1, absent.

48. Jugal, caudal process (Cabreira *et al*. 2016):

0, reaches the caudal margin of the ventral temporal fenestra;

1, does not reach the caudal margin of the ventral temporal fenestra.

49. Jugal; longitudinal ridge on the lateral surface of the body (Nesbitt 2011):

0, absent;

1, present.

50. Jugal; angle between ascending process and caudal process (Ezcurra 2006):

0, right or obtuse;

1, acute.

51. Jugal; total length relation relative to that of the skull (Cabreira *et al*. 2016):

0, more than 35%;

1, 35% or less.

52. Quadratojugal; dorsal ramus (Langer & Benton 2006):

0, longer than the cranial ramus;

1, equal or shorter than the cranial ramus.

53. Quadratojugal; angle between rostral and dorsal rami (Upchurch *et al*. 2007):

0, about 90° or more;

1, about 60° or lower.

54. Quadrate; ventral portion (Rauhut 2003):

0, aligned to the long axis of the bone;

1, caudally displaced relative to the long axis of the bone.

55. Postparietal (Jull 1994):

0, present;

1, absent.

56. Postemporal opening (Sereno & Novas 1994):

0, present minimally as a fissure;

1, absent.

57. Ectopterygoid; jugal process shape (Yates 2003):

0, slightly curved;

1, strongly curved and hook-shaped.

58. Basioccipital; blind pit in the (Nesbitt 2011; Müller *et al*. 2018):

0, absent;

1, one pit;

2, two pits.

59. Basipterygoid process, proximal part; angle to the proximal portion of the cultriform process (Butler *et al*. 2008):

0, less than 90°;

1, 90° or more than 90°.

60. Parabasisphenoid, foramina for entrance of cerebral branches of internal carotid artery into the braincase; position (Gower & Sennikov 1996):

0, on the ventral surface;

1, on the lateral surface.

61. Parabasisphenoid, lateral wall, caudoventral corner; semilunar depression, presence (Gower & Sennikov 1996):

0, present;

1, absent.

62. Basipterygoid process, shape (Cabreira *et al*. 2016):

0, rounded;

1, mediolaterally compressed.

63. Parabasisphenoid; median recess (Nesbitt 2011; Müller *et al*. 2018) ORDERED:

0, shallow depression;

1, fossa or deep depression.

64. Parabasisphenoid; caudal margin; outline in ventral view (Cabreira *et al*. 2016):

0, flat (approaching basal tubera);

1, excavated.

65. Opisthotic; paraoccipital process (Rauhut 2003):

0, directed laterally or dorsolaterally;

1, directed ventrolaterally.

66. Opisthotic; ventral ramus (Nesbitt 2011):

0, extends further laterally or about the same as lateralmost edge of exoccipital in caudal view;

1, covered by the lateralmost edge of exoccipital in caudal view.

67. Paroccipital processes, proportions (Butler *et al*. 2008):

0, short and deep (height > 1/2 length);

1, elongate and narrow (height < 1/2 length).

68. Exoccipital; relative positions of the exits of the hypoglossal nerve XII (Nesbitt 2011):

0, aligned in a near rostrocaudally plane;

1, aligned sub vertically.

69. Supraoccipital, dorsal surface, rugose ridge along exoccipital contact (Müller *et al*. 2018):

0, absent;

1, present.

70. Foramen for trigeminal nerve and middle cerebral vein (Nesbitt 2011):

0, combined and undivided;

1, fully or partially divided.

71. Predentary bone (Sereno 1986):

0, absent;

1, present.

72. Dentary; rostral portion (Nesbitt 2011; Cabreira *et al*. 2016):

0, rounded;

1, tapers to a sharp point.

73. Dentary; length versus height (Yates 2007):

0, less than 0.2;

1, more than 0.2.

74. Dentary; rostral tip, dorsal surface (Sereno 1999):

0, at nearly the same plane as the rest of the alveolar margin of the bone;

1, ventrally inclined.

75. Dentary; extension of the caudoventral process (Smith *et al*. 2007):

0, elongated, extends caudally to the caudodorsal process;

1, short, does not extend caudally to the caudodorsal process.

76. Dentary; coronoid process dorsally expanded (Sereno 1986):

0, absent;

1, present.

77. Dentary; Mandibular buccal emargination (Langer & Benton 2006):

0, absent, lateral dentary surface smooth;

1, present, lateral dentary surface with a caudal crest bordering an emargination which encompasses half of the dentary width.

78. Splenial; milohyoyd foramen (Rauhut 2003):

0, absent;

1, present.

79. Surangular; lateral surface (Tykoski & Rowe 2004):

0, evenly convex;

1, bears a prominent horizontal shelf.

80. Mandible; articular glenoid location (Gauthier 1986):

0, at the dorsal margin of the dentary;

1, well ventral of the dorsal margin of the dentary.

81. Articular, retroarticular process (Yates 2003):

0, shorter than the height of the mandible ventral to the glenoid;

1, longer than the height of the mandible ventral to the glenoid.

82. Retroarticular process, upturn (Baron *et al*. 2017) ORDERED:

0, present and strong, retroarticular forms nearly a right angle with the rest of the mandible;

1, present and subtle, retroarticular is slightly upturned at its distal end;

2, absent, retroarticular extends straight out from the caudal part of the mandible, or turns slightly downward

83) Mandibular fenestra anteroposterior length (modified from Butler, 2005) ORDERED

0) more than maximum depth of dentary ramus

1) less than maximum depth of dentary ramus (reduced)

2) completely closed (absent)

84. Premaxilla; teeth number (Nesbitt 2011) ORDERED:

0, three;

1, four;

2, five or more.

85. Premaxillary teeth, serration in the mesial margin (Rowe 1989):

0, present;

1, absent.

86. Premaxilla; tooth row (Tykoski & Rowe 2004):

0, extends ventrally below the internal narial fenestra;

1, does not extend caudally farther than the cranial edge of the internal narial fenestra.

87. Maxilla; teeth number (Nesbitt *et al*. 2009) ORDERED:

0, 15 or less;

1, more than 15 but less than 20;

0, 20 or more.

88. Teeth at the caudal half of maxilla/dentary; caudal edge (Nesbitt 2011):

0, concave or straight;

1, convex.

89. Middle maxillary/dentary teeth; serrations (Irmis *et al*. 2007) ORDERED:

0, small (c. 10 denticles per mm) forming right angles with the tooth margin;

1, large (c. 5 denticles per mm) but mostly forming right angles with the tooth margin;

2, larger forming oblique angles with the margin of the tooth.

90. Maxillary/dentary teeth; extensive planar wear facets across multiple teeth (Weishampel & Witmer 1990):

0, absent;

1, present.

91. Maxillary/dentary teeth; medial or lateral overlap of adjacent crowns (Sereno 1986):

0, absent;

1, present.

92. Maxillary/dentary teeth; some with moderately developed lingual expansion of crown cingulum (Sereno 1986):

0, absent;

1, present.

93. Maxillary/dentary teeth; crown shape (Sereno 1986):

0, apicobasally tall and blade like;

1, apicobasally short and subtriangular.

94. Middle maxillary/dentary teeth, distal margin (Sereno 1986):

0, never or subtly expanded at the base;

1, clearly expanded at the base.

95. Middle maxillary/dentary teeth; long axis (Cabreira *et al*. 2016):

0, caudally curved;

1, straight.

96. Dentary teeth; number (Smith *et al*. 2007):

0, maximal of 25;

1, more than 25.

97. Dentary teeth; rostral portion of the bone (Kammerer *et al*. 2012):

0, teeth remain relatively same size throughout rostral portion of dentition;

1, teeth significantly decrease in size rostrally.

98. Dentary teeth; rostral portion of the bone, long axis (Kammerer *et al*. 2012):

0, vertical;

1, inclined rostrally.

99. Marginal teeth, crown shape in distal-medial view (Sereno 1986):

0, blade-like;

1, labiolingually expanded.

100. Tooth implantation, teeth ankylosed into the alveoli (ankylothecodont) (Gauthier 1984; Baron *et al*. 2017):

0, absent (free at the base of tooth);

1, present.

101. Pterygoid teeth on palatal process (Rauhut, 2003):

0, present;

1, absent.

102. Axis; dorsal margin of the neural spine (Nesbitt 2011):

0, expanded caudodorsally;

1, arcs dorsally where the cranial portion height is equivalent to the caudal height.

103. Axis; parapophysis development (Tykoski & Rowe 2004):

0, well developed;

1, reduced.

104. Axis; epipophysis on postzygapophysis (Rauhut, 2003):

0, absent;

1, present.

105. Cervical vertebrae 3-5, centrum length (Sereno 1991):

0, shorter or the same length as the mid dorsal;

1, longer than mid dorsal.

106. Cervical vertebrae; deep recesses on the cranial face of the neural arch lateral to the neural canal (Nesbitt 2011):

0, absent;

1, present.

107. Third cervical vertebra; centrum length (Gauthier 1986):

0, subequal to the axis centrum;

1, longer than the axis centrum.

108. Cervical vertebrae; neural spine shape (Yates 2007):

0, not twice as long (at the midheight) as height;

1, at least twice at midheight as long as height.

109. Cranial cervical vertebrae, caudal chonos (Langer & Benton 2006) ORDERED:

0, absent;

1, as a shallow fossa;

2, as a deep excavation with a lamina covering the rostral extent.

110. Post-axial cranial cervical vertebrae; epipophyses (Gauthier 1986):

0, absent;

1, present.

111. Cervical vertebrae 6-9; epipophyses (Sereno *et al*. 1993):

0, absent;

1, present.

112. Cervical vertebrae; cranial portion of the centrum, pneumatic features/pleurocoels (Holtz 1994):

0, absent;

1, present.

113. Cervical vertebrae; neural arch (Galton & Upchurch 2004):

0, neural arch (from the base of neural canal to the top of postzygapophysis) higher than caudal articular facet of the centrum;

1, neural arch lower than caudal articular facet of the centrum.

114. Cervical vertebrae; middle portion of the ventral keel (Nesbitt 2011):

0, dorsal to the ventralmost extent of the centrum rim;

1, extends ventral to the centrum rims.

115. Cervical ribs; length of relative to the centrum (Tykoski & Rowe 2004):

0, no more than twice longer;

1, at least twice longer.

116. Presacral vertebrae; parapophysis position (Langer & Benton 2006):

0, parapophyses do not contact centrum in vertebra caudal to the twelfth presacral;

1, parapophyses contact centrum in vertebra caudal to the twelfth presacral.

117. Caudal cervical and/or dorsal vertebrae; hyposphene-hypantrum accessory articulations (Gauthier 1986):

0, absent;

1, present.

118. Trunk vertebrae; neural spine distal lateromedial expansion (Langer, 2004):

0, absent;

1, present.

119. Trunk vertebrae; neural spine, distal craniocaudal length (Bittencourt *et al*. 2014):

0, at least twice longer than the base;

1, less than twice longer than the base.

120. Trunk vertebrae; crest onto the lateral wall of caudal chonos (Yates 2004):

0, absent;

1, present.

121. Trunk vertebrae; caudal trunk centra; shape (Rauhut 2003):

0, short, centra are significantly shorter than high;

1, centra are approximately as long as high, longer than high.

122. Trunk vertebrae; transverse process shape (Tykoski & Rowe 2004):

0, subretangular;

1, subtriangular.

123. Trunk vertebrae; infradiapophyseal fossa (Yates 2007):

0, absent;

1, present.

124. Sacral centra (Nesbitt 2011):

0, separate;

1, co-ossified at the ventral edge.

125. Sacral neural spines; fused (Baron *et al*. 2017):

0, absent;

1, present.

126. Sacral vertebrae; incorporation of trunk vertebrae (Sereno *et al*. 1993):

0, free from the sacrum;

1, incorporated into the sacrum with ribs transverse processes articulating with the pelvis.

127. Sacral vertebrae, incorporation of caudal vertebrae (Galton 1976):

0, free from the sacrum;

1, incorporated into the sacrum with ribs transverse processes articulating with the pelvis.

128. Number vertebra fully incorporated to the sacrum (Gauthier 1986):

0, two;

1, three or more.

129. Sacral ribs (Nesbitt 2011):

0, almost entirely restricted to a single sacral vertebra;

1, shared between two sacral vertebrae.

130. First primordial sacral vertebra; articular surface of the rib (Langer & Benton 2006):

0, circular;

1, C-shaped in lateral view.

131. Sacral transverse process; development (Langer & Benton 2006):

0, craniocaudally short not roofing the space between ribs;

1, craniocaudally long, roofing the space between ribs.

132. Sacral ribs depth (Langer & Benton 2006):

0, as deep as half of the medial ilium depth;

1, deeper than half of the ilium depth.

133. Sacral rib and transverse process; lateral notch between elements (Bittencourt *et al*. 2014):

0, absent;

1, present.

134. First three caudal vertebrae; orientation of the neural spine (Langer & Benton 2006):

0, caudally inclined;

1, vertical.

135. Middle caudal centra; length (Yates 2003):

0, centra longer than twice the height of the cranial articular facet;

1, centra shorter than twice the height of the cranial articular facet.

136. Distal caudal vertebrae; prezygapophyses (Gauthier 1986):

0, length is less than a quarter of the adjacent centrum;

1, elongated, more than a quarter of the adjacent centrum.

137. Scapula; cranial margin (Nesbitt 2011):

0, straight convex or partially concave;

1, markedly concave.

138. Scapula; blade height (Sereno 1999):

0, less than 3 times distal width;

1, more than 3 times distal width.

139. Coracoid; caudal margin (Nesbitt 2011):

0, continuous (subcircular in lateral view);

1, with notch ventral to the glenoid.

140. Coracoid; post glenoid process (Nesbitt 2011):

0, absent or short;

1, extending caudal to glenoid.

141. Limbs; humerus+radius/femur+tibia length ratio (Gauthier 1984):

0, more than 0.55;

1, less than 0.55.

142. Humerus; apex of deltopectoral crest, situated at a point corresponding to (Bakker & Galton 1974) ORDERED:

0, about or less than 30% down the length of the humerus;

1, between 30% and 43% down the length of the humerus;

2, about or more than 43% down the length of the humerus.

143. Humerus; deltopectoral crest size (Yates 2007):

0, low;

1, expanded.

144. Humerus; deltopectoral crest shape (Yates 2007):

0, low rounded crest;

1, subtriangular, concave between apex and articulation;

2, subretangular, convex/straight between apex and articulation.

145. Limbs; humerus/femur length ratio (Novas 1996):

0, humerus longer than or subequal to 0.6 of the length of the femur;

1, humerus shorter than 0.6 of the length of the femur.

146. Humerus; distal end width (Langer & Benton 2006):

0, narrower or equal to 30% of humerus length;

1, wider than 30% of humerus length.

147. Humerus; shape in lateral view (Rauhut, 2003):

0, straight;

1, sigmoid.

148. Forelimb; humerus/radius length ratio (Langer & Benton 2006):

0, radius longer than 80% of humerus length;

1, radius shorter than or subequal to 80% of humerus length.

149. Ulna; olecranon process (Wilson & Sereno 1998):

0, short;

1, enlarged and strongly striated.

150. Distal carpal 1; articulation (Rauhut 2003) ORDERED:

0, does not articulate with metacarpal II;

1, articulates to metacarpal II.

2, caps metacarpal II.

151. Distal carpals; number of ossified elements (Sereno 1999):

0, five;

1, four.

152. Medialmost distal carpal; size (Gauthier 1986):

0, subequal other distal carpals;

1, significantly larger than other distal carpals.

153. Manus; length measured as the average length of digits I-III (Gauthier 1986):

0, accounts for less than 0.4 of the total length of humerus plus radius;

1, more than 0.4 of the total length of humerus plus radius.

154. Metacarpals; proximal ends (Sereno & Wild 1992):

0, overlap one another;

1, about one another without overlapping.

155. Manus; ungual phalanges (Gauthier 1986):

0, blunt unguals on at least digits II and III;

1, trenchant unguals on digits I to III.

156. Metacarpals II-III; extensor pits on distal/dorsal portion (Sereno *et al*. 1993):

0, absent or shallow;

1, deep.

157. Digit I; metacarpal/ungual phalanx length ratio (Sereno 1999):

0, metacarpal subequal or longer than ungual;

1, metacarpal shorter than ungual.

158. Metacarpal I; width (at the middle of the shaft)/length ratio (Bakker & Galton 1974):

0, width less than 0.35 of the length of the bone;

1, width more than 0.35 of the length of the bone.

159. Metacarpal I; distal condyles (Bakker & Galton 1974):

0, approximately aligned or slightly offset;

1, lateral condyle strongly distally expanded relative to medial condyle.

160. Manual digit I; first phalanx, twisting of the transverse axis (Sereno 1999):

0, not twisted;

1, twisted.

161. Manual digit I; first phalanx (Gauthier 1986):

0, not the longest non ungual phalanx of the manus;

1, longest non ungual phalanx of the manus.

162. Manual unguals; digit I & II length ratio (Yates 2007):

0, ungual of digit II as long as or longer than that of I;

1, ungual of digit II shorter than that of I.

163. Metacarpals; length ration between II and III (Gauthier 1986):

0, metacarpal II shorter than metacarpal III;

1, metacarpal II equal to or longer than metacarpal III.

164. Manual digit II; pre-ungual phalanx and phalanx 1 length ratio (Rauhut 2003):

0, pre-ungual phalanx equal or shorter;

1, pre-ungual phalanx longer.

165. Metacarpals; width (at midlength) ratio between elements II & III (Rauhut 2003):

0, equal;

1, metacarpal III at least 30% narrower.

166. Manual digit IV; number of phalanges (Gauthier 1986) ORDERED:

0, three or more;

1, two;

2, one or none.

167. Metacarpal IV; shaft width (Sereno *et al*. 1993):

0, about the same width as that of metacarpals I-III;

1, significantly narrower than that of metacarpals I-III.

168. Manual digit V; phalanges (Bakker & Galton 1974):

0, one or more phalanges;

1, no phalanges.

169. Manual Digit V (Bakker & Galton 1974):

0, present;

1, absent.

170. Ilium; preacetabular ala, tip shape (Galton 1976):

0, pointed, with vertex projected cranially;

1, rounded;

2, squared.

171. Ilium; preacetabular ala length (Galton 1976):

0, does not extend cranial to the cranial margin of the pubic peduncle;

1, extends cranial to the cranial margin of the pubic peduncle.

172. Ilium; supraacetabular crest, position of thicker (lateromedially) portion (Yates 2003):

0, at the center of the acetabulum;

1, closer to pubic peduncle.

173. Ilium; supraacetabular crest, extension on pubic peduncle (Nesbitt *et al*. 2009):

0, ends before the distal margin of the peduncle;

1, extends along the peduncle length.

174. Ilium; fossa for the attachment of m. caudifemoralis brevis (Gauthier & Padian, 1985) ORDERED:

0, absent;

1, present as an embankment on the lateral side of the caudal portion of the ilium;

2, present as a fossa on the ventral surface of postacetabular part of ilium.

175. Ilium; ventral margin of the acetabular wall (Bakker & Galton 1974) ORDERED:

0, convex;

1, slightly concave, straight or slightly convex;

2, markedly concave.

176. Ilium; acetabular antitrochanter (Sereno & Arcucci 1994):

0, absent or just a slightly planar surface;

1, raised shelf.

177. Ilium; brevis fossa lateral wall (Smith *et al*. 2007):

0, vertical;

1, lateroventrally directed.

178. Ilium; pubic peduncle distal articulation (Smith *et al*. 2007):

0, not expanded;

1, expanded distally.

179. Ilium; ischiadic peduncle orientation in lateral view (Langer & Benton 2006):

0, mainly vertical;

1, well expanded caudal to the cranial margin of the postacetabular embayment.

180. Ilium; strong pillar caudal to the preacetabular embayment (Nesbitt 2011):

0, absent;

1, present.

181. Ilium; dorsal margin shape (Gauthier 1986):

0, sigmoid;

1, entirely convex.

182. Ilium; position of dorsal margin concavity (Cabreira *et al*. 2016):

0, above the acetabulum;

1, caudally displaced.

183. Ilium; maximum length of the postacetabular ala (Yates 2007):

0, shorter than or subequal to the space between the pre and postacetabular embayments;

1, longer than the space between the pre and postacetabular embayments.

184. Ilium; pubic articulation orientation (Langer & Benton 2006) ORDERED:

0, ventral;

1, cranioventral;

2, cranial.

185. Ilium, pubic peduncle, shape; width (craniocaudal) at mid-length vs total length (Galton 1976):

0, less than 0.5;

1, 0.5 or more.

186. Ilium, pubic peduncle, position; angle of the long axis to that of the long axis of the iliac lamina (Cabreira *et al*. 2016) ORDERED:

0, more than 45 degrees;

1, about 45 degrees;

2, less than 45 degrees.

187. Ilium, dorsal lamina; depth relative to the acetabulum (Makovicky & Sues 1998):

0, shallow that two times;

1, two times deeper or more.

188. Pubis length (Novas 1996):

0, less than 70% or equal of femoral length;

1, more than 70% or more of femoral length.

189. Pubis, orientation (modified from Sereno, 1986) ORDERED

0) anteroventral (propubic), angle formed between iliac articular surface and main axis of the base of the shaft >75°.

1) ventral or slightly posteroventral (mesopubic), angle formed between iliac articular surface and main axis of the base of the shaft ≤75°.

2) rotated posteroventrally to lie alongside the ischium (opistopubic)

190. Pubis; distal end (Gauthier 1986) ORDERED:

0, unexpanded;

1, expanded relative to the shaft;

2, expanded and at least twice the breadth of the pubic shaft (pubis boot).

191. Pubis; medial articulation of the pair (Tykoski 2005):

0, complete, reaches the distal edge of the pubis;

1, forms a medial hiatus on the distal portion (bevel).

192. Pubis; distal apron (Langer & Benton 2006):

0, straight;

1, lateral portion flipped caudally.

193. Pubis; proximal portion (Nesbitt 2011):

0, articular surfaces with the ilium and the ischium continuous;

1, articular surfaces with the ilium and the ischium separated by a gap.

194. Pubis; shaft in lateral view (Harris, 1998):

0, cranially bowed;

1, straight.

195. Pelvis; ischio-pubis contact (Benton & Clark 1988):

0, present and extended ventrally;

1, present and reduced to a thin proximal contact.

196. Pubis; distal pubis mediolateral width (Galton 1976):

0, nearly as broad as proximal width of the bone;

1, significantly narrower than proximal width of the bone.

197. Ischium; medial contact with antimere (Novas 1996):

0, restricted to the ventral edge;

1, more dorsally extensive contact.

198. Ischium; dorsolateral sulcus (Yates 2003):

0, absent;

1, present.

199. Ischium; outline of the distal portion (Sereno 1999):

0, thin/plate-like;

1, rounded or elliptical;

2, subtriangular.

200. Ischium; distal portion (Smith & Galton 1990):

0, unexpanded;

1, expanded relative to the ischial shaft.

201. Ischium; proximal articular surfaces (Irmis *et al*. 2007) ORDERED:

0, continuous ilium and pubis articular surfaces;

1, ilium and pubis articular surfaces continuous but separated by a fossa;

2, ilium and pubis articular surfaces separated by a non-articulating concave surface.

202. Ischium; length relative to the dorsal margin of the iliac blade minus the preacetabular ala (Nesbitt 2011):

0, about the same length or shorter;

1, markedly longer.

203. Femur; proximal portion, craniomedial tuber (Gauthier 1986) ORDERED:

0, small/unprojected/rounded;

1, small and angled (separated from the caudomedial by the lig. sulcus);

2, offset medially or caudally relative to the caudomedial tuber.

204. Femur; ligament sulcus (Cabreira *et al*. 2016):

0, does not form a medial excavation in proximal view;

1, forms a medial excavation in proximal view.

205. Femur; proximal portion, caudomedial tuber (Novas 1996) ORDERED:

0, present and largest of the proximal tubera;

1, small;

2, absent.

206. Femur; proximal portion, craniolateral tuber (Sereno & Arcucci 1994):

0, absent (the craniolateral face is flat or equally rounded);

1, present.

207. Femur; medial articular surface of the head in dorsal view (Nesbitt 2011):

0, rounded;

1, flat/straight.

208. Femur; head, expansion/shaft transition (Sereno & Arcucci 1994) ORDERED:

0, smooth transition from the femoral shaft to the head;

1, kinked transition from the femoral shaft to the head;

2, kinked transition and expanded head.

209. Femur; head long axis angle to the distal intercondylar line (Benton & Clark 1988) ORDERED:

0, 45° or more;

1, 20-45°;

2, 0-20°.

210. Femur; head in medial and lateral views (Sereno & Arcucci 1994):

0, rounded;

1, hook shaped.

211. Femur; dorsolateral trochanter (Nesbitt 2011):

0, absent;

1, present.

212. Femur; “lesser” trochanter (Bakker & Galton 1974) ORDERED:

0, absent;

1, present and forms a steep margin with the shaft but is completely connected to it;

2, present and separated from the shaft by a marked cleft;

3, present and approaches the proximal articulation of the bone.

213. Femur; medial articular facet of the proximal portion in caudomedial view (Nesbitt 2011):

0, rounded;

1, straight.

214. Femur; craniolateral surface of the femoral head (Sereno & Arcucci 1994):

0, smooth, featureless;

1, ventral emargination present.

215. Femur; “trochanteric shelf” (Gauthier 1986):

0, absent;

1, present.

216. Femur; head, facies articularis antitrochanterica (Novas 1996):

0, level with “greater trochanter”;

1, ventrally descended.

217. Femur; “greater trochanter” shape (Sereno 1999):

0, rounded;

1, angled.

218. Femur; transverse groove on proximal surface (Ezcurra 2006):

0, absent;

1, present.

219. Femur; “lesser” trochanter, lateromedial position (Yates 2007):

0, closer to the medial edge;

1, closer to the lateral margin.

220. Femur; fourth trochanter shape (Gauthier 1986):

0, mound-like or subtle crest;

1, flange.

221. Femur; fourth trochanter; symmetry (Langer & Benton 2006):

0, symmetrical, distal and proximal margins forming similar low angle slopes to the shaft;

1, asymmetrical, distal margin forming a steeper angle to the shaft.

222. Femur; bone wall. thickness at or near mid shaft (Nesbitt 2011):

0, thickness diameter > 0.3;

1, thin/thickness diameter >0.2, <0.3.

223. Femur; distal condyles, extension of the caudal division (Nesbitt 2011):

0, less than 1/4 the length of the shaft;

1, between 1/4 and 1/3 the length of the shaft.

224. Femur cranial surface of the distal portion (Nesbitt *et al*. 2009):

0, smooth;

1, distinct scar orientated mediolaterally.

225. Femur; crista tibiofibularis size/shape (Sereno & Arcucci 1994) ORDERED:

0, smaller in size to the lateral condyle;

1, larger or equal than the lateral condyle;

2, larger/equal and globular.

226. Femur; craniomedial corner of the distal end (Nesbitt *et al*. 2009):

0, rounded;

1, squared off near 90° or acute.

227. Femur; cranial margin in distal view (Cabreira *et al*. 2016):

0, concave;

1, straight or convex.

228. Femur, distal end, sharp ridge on the anteromedial edge (Martínez *et al*. 2016):

0, absent;

1, present.

229. Femur, distal end, lateral tuberosity on the anterolateral edge (Martínez *et al*. 2016):

0, absent;

1, present.

230. Femur, distal end, wide concavity on the posterolateral surface of the crista tibiofibularis (Martínez *et al*. 2016):

0, absent;

1, present.

231. Femur, surface between the lateral condyle and crista tibiofibularis on the distal surface (Nesbitt 2011):

0, smooth;

1, deep groove.

232. Hindlimb; tibia or fibula relative length to the femur (Gauthier 1986):

0, femur longer or about the same length as the tibia/fibula;

1, tibia/fibula longer than femur.

233. Tibia; depth of the sulcus lateral to cnemial crest (Langer *et al*. 2011):

0, no deeper than 10% of the length of proximal surface of tibia;

1, more than 10% of the length of proximal surface of tibia.

234. Tibia; cnemial crest (Benton & Clark 1988) ORDERED:

0, absent or just a slight bump;

1, present and straight;

2, present arcs craniolaterally.

235. Tibia; proximal portion, fibular condyle (Langer & Benton 2006) ORDERED:

0, offset cranially from the medial condyle;

1, level with the medial condyle at its caudal border;

2, displaced caudally.

236. Tibia lateral side of the proximal portion (Gauthier 1986) ORDERED:

0, smooth or scared;

1, dorsoventrally oriented crest present;

2, well-developed ridge.

237. Tibia; separation of the proximal condyles (Rauhut 2003):

0, separated by a shallow notch;

1, separated by a deep groove.

238. Tibia; separation of the condyles in proximal view (Cabreira *et al*. 2016):

0, single notch/groove;

1, two separated notches.

239. Tibia; distal portion, caudolateral flange (Novas 1992) ORDERED:

0, absent;

1, present;

2, present and extends well lateral to the craniolateral corner.

240. Tibia; distal end, caudal margin shape (Irmis *et al*. 2007):

0, straight or convex;

1, concave.

241. Tibia; distal surface outline (Rauhut 2003):

0, rounded or subquadrangular (approximately as wide as long);

1, mediolaterally expanded.

242. Tibia; distal portion; caudomedial surface (Nesbitt 2011):

0, rounded surface;

1, distinct proximodistally oriented ridge present.

243. Tibia; distal portion, lateral side (Novas 1996):

0, smooth rounded;

1, proximodistally oriented groove.

244. Tibia; distal surface, caudomedial notch (Yates 2007):

0, absent;

1, present.

245. Tibia; astragalar articulation (Novas 1996):

0, tibia articulates with astragalus medially to the ascending process;

1, tibia covers the medial and caudal portion of the dorsal surface of astragalus.

246. Astragalus; caudal margin, dorsally expanded process (Sereno & Arcucci 1994):

0, absent or poorly expanded;

1, expanded into a distinct raised process caudal to ascending process.

247. Astragalus; proximal margin in caudal view (Cabreira *et al*. 2016):

0, straight at the lateralmost portion;

1, depressed at the lateralmost portion (with subtle raised margin medial to it).

248. Astragalus; cranial ascending process (Gauthier 1986):

0, absent;

1, present.

249. Astragalus; tibial articulation caudal to the ascending process (Langer & Benton 2006):

0, continuous to the medial articulation surface;

1, markedly rimmed and elliptical fossa (separated by a ridge or step from the medial surface).

250. Astragalus; proximal articular facet for fibula (Langer & Benton 2006) ORDERED:

0, equal more than 0.3 of the transverse width of the bone;

1, less than 0.3 of the transverse width of the bone;

2, vertical (no horizontal platform).

251. Astragalus; caudal groove (Nesbitt 2011):

0, present;

1, absent.

252. Astragalus; shape of the craniomedial margin (Yates 2007):

0, obtuse or forming a right angle;

1, acute.

253. Astragalus; medial portion of the tibial facet (Benton 1999):

0, concave or flat;

1, divided into caudomedial and craniolateral basins.

254. Astragalus; shape (Cabreira *et al*. 2016):

0, more than 80% broad lateromedially than craniocaudally;

1, less than 80% broad craniocaudally than lateromedially.

255. Astragalus; cranial margin (Cabreira *et al*. 2016):

0, straight or concave;

1, deeply excavated with a groove extending along the distal surface of the bone.

256. Astragalus; cranial ascending process, cranial margin (Langer 2004) ORDERED:

0, continuous to the cranial surface of the astragalar body;

1, separated from the cranial surface of the astragalar body by an oblique slope;

2, separated from the cranial surface of the astragalar body by a platform.

257. Astragalus; caudolateral process (Cabreira *et al*. 2016):

0, continuous to the caudal margin of the bone;

1, displaced cranially.

258. Astragalus-calcaneum; articulation (Sereno & Arcucci 1994):

0, free;

1, co-ossified.

259. Calcaneum; calcaneal tuber (Gauthier 1986):

0, present;

1, absent.

260. Calcaneum; tibial articulation (Langer *et al*. 2011):

0, absent;

1, present.

261. Calcaneum; articular surface for the fibula (Novas 1996):

0, convex;

1, concave.

262. Calcaneum; shape (Langer & Benton 2006):

0, proximodistally compressed and subtriangular, with short caudal projection and medial processes;

1, transversely compressed and subretangular, reduced projection and processes.

263. Distal tarsals; fused to proximal ends of metatarsals (Baron *et al*. 2017):

0, absent;

1, present.

264. Distal tarsal 3; articulation with metatarsus (Butler *et al*. 2008):

0, articulates with metatarsal III only;

1, articulates with metatarsal II and III.

265. Distal tarsal 4; caudal prong (Langer & Benton 2006):

0, blunt;

1, pointed.

266. Distal tarsal 4; medial process (Nesbitt 2011):

0, absent;

1, distinct in the craniocaudal middle of the element.

267. Distal tarsal 4; proximal surface (Sereno & Arcucci 1994):

0, flat;

1, distinct proximally raised region on the caudal portion.

268. Metatarsus; maximum length (Benton 1999):

0, equal or shorter than 50% of tibial length;

1, longer than 50% of tibial length.

269. Metatarsus; metatarsals I and II, articulation (Gauthier 1986):

0, Metatarsal I reaches the proximal surface of metatarsal II;

1, Metatarsal I does not reach and attaches onto the medial side of metatarsal II.

270. Metatarsus; metatarsals II and IV; length relation (Gauthier 1986):

0, Metatarsal IV longer than metatarsal II;

1, Metatarsal IV subequal or shorter than metatarsal II.

271. Metatarsal IV; distal articulation surface (Sereno 1999):

0, broader than deep to as broad as deep;

1, deeper than broad.

272. Metatarsal IV; proximal portion (Sereno 1999):

0, narrow;

1, expanded, overlapping the cranial surface of metatarsal V.

273. Metatarsal IV; shape (Novas 1996):

0, straight;

1, laterally curved at the distal end.

274. Metatarsus; metatarsals III and V; length relation (Carrano *et al*. 2002):

0, Metatarsal V equal to or longer than 50% of metatarsal III;

1, Metatarsal V shorter than 50% of metatarsal III.

275. Metatarsal V; proximal (Yates 2003) ORDERED:

0, unexpanded;

1, expanded;

2, expanded, with a width more than 30% the length of the bone.

276. Metatarsal V; distal tip (Gauthier 1984):

0, blunt and with phalanges;

1, without phalanges and tapers to a point.

277. Osteoderms; dorsal to the vertebral column (Gauthier 1984):

0, absent;

1, present.

278. Palpebral (Nesbitt 2011):

0, absent;

1, present.

279. Palpebral position (Butler *et al*. 2008):

0, above orbit;

1, projects into orbit.

280. Femur, fourth trochanter orientation (Butler *et al*. 2008):

0, perpendicular to axis;

1, pendant.

281. Tibia, distal condyles (Baron *et al*. 2017):

0, condyles equally extensive;

1, medial condyle extends further distally;

2, lateral condyle extends further.

282. Dentary, anterior half, position of the Meckelian groove: (Nesbitt 2011):

0, dorsoventral center of the dentary;

1, restricted to the ventral border.

283. Ilium, pubic peduncle ventral extension (Novas *et al*. 2021):

0, level with ischiadic peduncle

1, significantly more ventrally extended than the ischiadic peduncle

284. Premaxilla, height-length ratio below external naris (Novas *et al*. 2021):

0, 0.5-1.25

1, <0.5

2, >1.25

285. Exoccipital, lateral surface (Novas *et al*. 2021):

0, without subvertical crest (= metotic strut)

1, with clear crest (= metotic strut) lying anterior to both external foramina for hypoglossal nerve (XII)

2, with clear crest (= metotic strut) present anterior to the more posterior external foramina for hypoglossal nerve (XII)

286. Skull, Antorbital fossa (Novas *et al*. 2021):

0, restricted to the lacrimal and dorsal process of the maxilla

1, present on the lacrimal, dorsal process of the maxilla, and the dorsolateral margin of the posterior process of the maxilla (the ventral border of the antorbital fenestra)

287. Pubis, expanded distal margin (Novas *et al*. 2021):

0, mediolaterally thick and rounded

1, thin

288. Scapula, distal end of the blade (Novas *et al*. 2021):

0, distinctly anteroposteriorly expanded

1, poorly anteroposteriorly expanded, without an increased divergence between the anterior and posterior margins with respect to the rest of the blade

289. Metacarpus, metacarpal IV x metacarpal III lengths ratio (Novas *et al*. 2021):

0, equal or more than 0.6

1, less than 0.6

290. Manual digits, unguals of digits II-IV length (Nesbitt *et al*. 2015; Ezcurra 2016; Novas *et al*. 2021):

0, about the same length or shorter than the last phalanx of the same digit

1, distinctly longer than the last phalanx of the same digit

291. Maxilla, tooth row inset medially from lateral surface (Garcia *et al*. 2024): ORDERED

0, absent

1, present

292. Dorsal vertebrae, anterior centra (first three vertebrae) (Garcia *et al*. 2021):

0, about as long as tall or shorter than tall

1, significantly longer than tall

283. Pubis, anteriorly expanded distal end (new):

0, absent

1, present

**2. Modifications to previous versions of the phylogenetic data matrix**

Character 190:
*Macrocollum itaquii* changed from (0) to (1)

*Coelophysis bauri* changed from (2) to (1)

Character 191:
*Herrerasaurus ischigualastensis* changed from (0) to (1)

*Sanjuansaurus gordilloi* changed from (0) to (1)

*Gnathovorax cabreirai* changed from (0) to (1)

Character 192:
*Asilisaurus kongwe* changed from (1) to (0)

Character 190:
*Macrocollum itaquii* changed from (0) to (1)

Character 287:
*Eodromaeus murphi* changed from (?) to (0)
*Macrocollum itaquii* changed from (?) to (0)

*Buriolestes schultzi* changed from (?) to (0)

**3. References for supplementary information**

Bakker, R. T., and Galton, P. M. (1974). Dinosaur monophyly and a new class of vertebrates. Nature 248, 168–172.

Baron, M. G., Norman, D. B., Barrett, P. M. 2017 A new hypothesis of dinosaur relationships and early dinosaur evolution. Nature 543, 501–506.

Benton, M. J. (1999). *Scleromochlus taylori* and the origin of dinosaurs and pterosaurs. Philos. Trans. R. Soc. Lond. B Biol. Sci. 354, 1423–1446

Benton, M. J., and Clark, J. M. (1988). Archosaur phylogeny and the relationships of the Crocodylia. In the Phylogeny and Classification of the Tetrapods. Vol. **1**: Amphibians and Reptiles, M. J. Benton ed. (Oxford: Clarendon Press), pp. 295–338.

Bittencourt, J. S., Arcucci, A. B., Marsicano, C. A., and Langer, M. C. (2014). Osteology of the Middle Triassic archosaur *Lewisuchus admixtus* Romer (Chañares Formation, Argentina), its inclusivity, and relationships among early dinosauromorphs. J. Syst. Palaeontol. **13**, 189–219.

Butler, R. J., Upchurch, P., Norman, D. B. (2008). The phylogeny of ornithischian dinosaurs. J. Syst. Palaeontol. 6, 1–40.

Butler, R. J. (2005). The ‘fabrosaurid’ ornithischian dinosaurs of the upper Elliot Formation (Lower Jurassic) of South Africa and Lesotho. Zool. J. Linn. Soc. 145, 175–218.

Cabreira, S. F. et al. (2016). A unique Late Triassic dinosauromorph assemblage reveals dinosaur ancestral anatomy and diet. Curr. Biol*.* 26, 3090–3095.

Carrano, M. T., Sampson, S. D., and Forster, C. A. (2002). The osteology of *Masiakasaurus knopfleri*, a small abelisauroid (Dinosauria: Theropoda) from the Late Cretaceous of Madagascar. J. Vert. Paleontol. 22, 510–534.

Ezcurra, M. D. (2006). A review of the systematic position of the dinosauriform archosaur *Eucoelophysis baldwini* Sullivan & Lucas, 1999 from the Upper Triassic of New Mexico, USA. Geodiversitas 28, 649–684.

Galton, P. M. (1976). Prosauropod dinosaurs (Reptilia:Saurischia) of North America. Postilla 169, 1–98.

Galton, P. M., and Upchurch, P. (2004). Basal Sauropodomorpha-Prosauropoda. In the Dinosauria 2nd edn, D. B. Weishampel, P. Dodson, H. Osmolska. ed. (Berkeley: Univ. of California Press), pp. 232–258.

Garcia, M. S., Cabreira, S. F., da Silva, L. R., Pretto, F. A., & Müller, R. T. (2024). A saurischian (Archosauria, Dinosauria) ilium from the Upper Triassic of southern Brazil and the rise of Herrerasauria. The Anatomical Record, 307(4), 1011–1024.

Garcia, M. S., Müller, R. T., Pretto, F. A., Da-Rosa, Á. A., & Dias-Da-Silva, S. (2021). Taxonomic and phylogenetic reassessment of a large-bodied dinosaur from the earliest dinosaur-bearing beds (Carnian, Upper Triassic) from southern Brazil. Journal of Systematic Palaeontology, 19(1), 1–37.

Gauthier, J. A. (1984). PhD thesis, University of California, Berkeley.

Gauthier, J. A. (1986). Saurischian monophyly and the origin of birds. Mem. Calif. Acad. Sci. 8,1–55.

Gauthier, J. A., and Padian, K. (1987). Phylogenetic, functional, and aerodynamic analyses of the origin of birds and their flight. In the Beginnings of Birds: Proceedings of the International Archaeopteryx Conference, M. K., Hecht, J. H. Ostrom, G. Viohl, P. Wellnhofer ed. (Eichstätt: Freunde des Juras-Museums), pp. 185–197.

Gower, D. J., and Sennikov, A. G. (1996). Morphology and phylogenetic informativeness or early archosaur braincases. Palaeontology 39, 883–906.

Harris, J. D. (1998). A Reanalysis of *Acrocanthosaurus atokensis*, its Phylogenetic Status, and Paleobiogeographic Implications, Based on a New Specimen from Texas. New Mexico Mus. Nat. Hist. Sci. Bull. 13, 1–175.

Holtz, T. R. (1994). The phylogenetic position of the Tyrannosauridae: implications for theropod systematics. J. Paleontol. 68, 1100–1117.

Irmis, R. B., Nesbitt, S. J., Padian, K., Smith, N. D., Turner, A. H., Woody, D, and Downs, A. (2007). A Late Triassic dinosauromorph assemblage from New Mexico and the rise of dinosaurs. Science 317, 358–361.

Juul, L. (1994). The phylogeny of basal archosaurs. Paleontol. Afr. 31, 1–38.

Kammerer, C. F., Nesbitt, S. J., Shubin, N. H. (2012). The first silesaurid dinosauriform from the Late Triassic of Morocco. Acta Palaeontol. Pol. 57, 277–284.

Langer, M. C. (2004). Basal saurischian. In The Dinosauria 2nd edn, D. B. Weishampel, P. Dodson, H. Osmolska. ed. (Berkeley: Univ. of California Press), pp. 25–46.

Langer, M. C., and Benton, M. J. (2006). Early dinosaurs: A phylogenetic study. J. Syst. Palaeontol. 4, 309–358.

Langer, M. C., Bittencourt, J. S. Schultz,C. L. (2011). A reassessment of the basal dinosaur *Guaibasaurus candelariensis*, from the Late Triassic Caturrita Formation of south Brazil. Earth Environ. Sci. Trans. R. Soc. Edinb. 101, 301–332.

Makovicky, P. J., and Sues, H.-D. (1998). Anatomy and phylogenetic relationships of the theropod dinosaur *Microvenator celer* from the Lower Cretaceous of Montana. American Museum Novitates 3240, 1–27.

Martínez, R. N., Apaldetti, C., Correa, G. A., Abelín, D. (2016). A Norian lagerpetid dinosauromorph from the Quebrada del Barro Formation, northwestern Argentina. Ameghiniana 53, 1–13.

Martz, J. W., Small, B. J. (2019). Non-dinosaurian dinosauromorphs from the Chinle Formation (Upper Triassic) of the Eagle Basin, northern Colorado: *Dromomeron romeri* (Lagerpetidae) and a new taxon*, Kwanasaurus williamparkeri* (Silesauridae). PeerJ 7, e7551.

Müller, R. T., Langer, M. C., Dias-da-Silva, S. (2018) An exceptionally preserved association of complete dinosaur skeletons reveals the oldest long-necked sauropodomorphs. Biol Lett. 14(11):20180633

Nesbitt, S. J. (2011). The early evolution of Archosauria: relationships and the origin of major clades. B. Am. Mus. Nat. Hist. 352, 1–292.

Nesbitt, S. J., Langer, M. C., Ezcurra, M. D. (2019). The anatomy of *Asilisaurus kongwe*, a dinosauriform from the Lifua Member of the Manda Beds (~ Middle Triassic) of Africa. Anat. Rec

Nesbitt, S. J., Smith, N. D., Irmis, R. B., Turner, A. H., Downs, A., Norell, M. A. (2009). A complete skeleton of a Late Triassic saurischian and the early evolution of dinosaurs. Science 326, 1530–1533.

Novas, F. E. (1992). Phylogenetic relationships of the basal dinosaurs, the Herrerasauridae. Palaeontology 35, 51–62.

Novas, F. E. (1996). Dinosaur monophyly. J. Vert. Paleontol. 16, 723–741.

Novas, F. E., Agnolin, F. L., Ezcurra, M. D., Müller, R. T., Martinelli, A. G., & Langer, M. C. (2021). Review of the fossil record of early dinosaurs from South America, and its phylogenetic implications. Journal of South American Earth Sciences, 110, 103341.

Rauhut, O. W. M. (2003). The Interrelationships and Evolution of Basal Theropod Dinosaurs. Spec. Pap. Palaeontol. 69, 1–213.

Rowe, T. (1989). A new species of the theropod dinosaur *Syntarsus* from the Early Jurassic Kayenta Formation of Arizona. J. Vert. Paleontol. 9, 125–36.

Sereno, P. C. (1986). Phylogeny of the bird-hipped dinosaurs (Order Ornithischia). Natl. Geogr. Res. 2, 234–256.

Sereno, P. C. (1991). Basal archosaurs: phylogenetic relationships and functional implications. J. Vert. Paleont. 11.S4, 1–53.

Sereno, P. C. (1999). The evolution of dinosaurs. Science 284, 2137–2147.

Sereno, P. C., and Arcucci, A. B. (1994). Dinosaurian precursors from the Middle Triassic of Argentina: *Marasuchus lilloensis*, n. gen. J. Vert. Paleontol. 14, 53–73.

Sereno, P. C., and Novas, F. E. (1994). The skull and neck of the basal theropod *Herrerasaurus ischigualastensis*. J. Vert. Paleont, 13, 451–476.

Sereno, P. C., and Wild, R. (1992). *Procompsognathus*: theropod,“thecodont” or both?. J. Vert. Paleont. 12, 435–458.

Sereno, P. C., Forster, C. A., Rogers, R. R., Monetta, A. M. (1993). Primitive dinosaur skeleton from Argentina and the early evolution of Dinosauria. Nature 361, 64–66.

Sereno, P. C., Wilson, J. A., Larsson, H. C. E., Dutheil, D. B., Sues, H.-D. (1994). Early Cretaceous dinosaurs from the Sahara. Science 265, 267–271.

Smith, N. D., Makovicky, P. J., Hammer, W. R., Currie, P. J. (2007). Osteology of *Cryolophosaurus ellioti* (Dinosauria: Theropoda) from the Early Jurassic of Antarctica and implications for early theropod evolution. Zool. J. Linn. Soc. 151, 377–421.

Tykoski, R. (2005). PhD thesis, University of Texas, Austin.

Tykoski, R. S., and Rowe, T. (2004). Ceratosauria. In The Dinosauria 2nd edn, D. B. Weishampel, P. Dodson, H. Osmolska. ed. (Berkeley: Univ. of California Press), pp. 47–70.

Upchurch, P., Barrett, P. M., Galton, P. M. (2007). A phylogenetic analysis of basal sauropodomorph relationships: Implications for the origin of sauropod dinosaurs. Spec. Pap. Paleontol. 77, 57–90.

Weishampel, D. B., and Witmer, L. M. (2004). *Lesothosaurus*, *Pisanosaurus*, and *Technosaurus*. In the Dinosauria 2nd edn, D. B. Weishampel, P. Dodson, H. Osmolska. ed. (Berkeley: Univ. of California Press), pp. 416–425.

Wilson, J. A., and Sereno, P. C. (1998). Early evolution and higher-level phylogeny of sauropod dinosaurs. J.Vert. Paleontol. 18.S2, 1–79.

Yates, A. M. (2007). The first complete skull of the Triassic dinosaur *Melanorosaurus Haughton* (Sauropodomorpha: Anchisauria). Spec. Pap. Palaeontol. 77, 9–55.

Yates, A. M. (2004). *Anchisaurus polyzelus* (Hitchcock): the smallest known Sauropod Dinosaur and the evolution of gigantism among Sauropodomorph Dinosaurs. Postilla 230, 1–58.

Yates, A. M., and Kitching, J. W. (2003). The earliest known sauropod dinosaur and the first steps towards sauropod locomotion. Proc. R. Soc. Lond. B Biol. Sci. 270, 1753–1758.
